# Disease Control Implications of India's Changing Multi-Drug Resistant Tuberculosis Epidemic

**DOI:** 10.1371/journal.pone.0089822

**Published:** 2014-03-07

**Authors:** Sze-chuan Suen, Eran Bendavid, Jeremy D. Goldhaber-Fiebert

**Affiliations:** 1 Department of Management Science and Engineering, Stanford University Stanford, Stanford, California, United States of America; 2 Division of General Medical Disciplines, Department of Medicine, Stanford University Stanford, Stanford, California, United States of America; 3 Center for Health Policy and the Center for Primary Care and Outcomes Research, Stanford University, Stanford, California, United States of America; Johns Hopkins Bloomberg School of Public Health, United States of America

## Abstract

**Background:**

Multi-drug resistant tuberculosis (MDR TB) is a major health challenge in India that is gaining increasing public attention, but the implications of India's evolving MDR TB epidemic are poorly understood. As India's MDR TB epidemic is transitioning from a treatment-generated to transmission-generated epidemic, we sought to evaluate the potential effectiveness of the following two disease control strategies on reducing the prevalence of MDR TB: a) improving treatment of non-MDR TB; b) shortening the infectious period between the activation of MDR TB and initiation of effective MDR treatment.

**Methods and Findings:**

We developed a dynamic transmission microsimulation model of TB in India. The model followed individuals by age, sex, TB status, drug resistance status, and treatment status and was calibrated to Indian demographic and epidemiologic TB time trends. The main effectiveness measure was reduction in the average prevalence reduction of MDR TB over the ten years after control strategy implementation.

We find that improving non-MDR cure rates to avoid generating new MDR cases will provide substantial non-MDR TB benefits but will become less effective in reducing MDR TB prevalence over time because more cases will occur from direct transmission – by 2015, the model estimates 42% of new MDR cases are transmission-generated and this proportion continues to rise over time, assuming equal transmissibility of MDR and drug-susceptible TB. Strategies that disrupt MDR transmission by shortening the time between MDR activation and treatment are projected to provide greater reductions in MDR prevalence compared with improving non-MDR treatment quality: implementing MDR diagnostic improvements in 2017 is expected to reduce MDR prevalence by 39%, compared with 11% reduction from improving non-MDR treatment quality.

**Conclusions:**

As transmission-generated MDR TB becomes a larger driver of the MDR TB epidemic in India, rapid and accurate MDR TB diagnosis and treatment will become increasingly effective in reducing MDR TB cases compared to non-MDR TB treatment improvements.

## Introduction

Worldwide, tuberculosis (TB) prevalence has declined by over 30% since 1990 [1]. However, selective pressures from the increased use of TB medications have led to the emergence and growth of multi-drug resistant (MDR) TB, defined as strains of *Mycobacterium tuberculosis* resistant to at least isoniazid and rifampin, two first line anti-TB medications. Strains resistant to no more than one of these medications are referred to as non-MDR TB [1]–[4]. Drug resistance challenges TB control, as diagnostic technology to identify drug resistance is often unavailable, mortality rates are high, and MDR TB treatments are more lengthy, toxic, and costly [5]. Improving non-MDR TB treatment could provide an effective approach to MDR control as long as most incident MDR TB cases develop during treatment of non-MDR TB. However, if MDR TB prevalence increases sufficiently, transmission-generated disease could eventually account for the majority of incident MDR TB, as has been observed in South Africa and China [6], [7].

The challenge of addressing TB and MDR TB is critical for India, home to over 25% of the world's TB cases [1]. In 1997, India's Revised National Tuberculosis Control Programme (RNTCP) implemented the World Health Organization's (WHO) Directly Observed Treatment Short-Course (DOTS) strategy. Treatment success rates have improved since then, but effective TB control remains challenged by treatment provided outside of RNTCP and imperfect treatment completion rates, which can generate new MDR TB cases [8], [9]. Private clinics in India are often used by patients seeking TB treatment and may employ treatment regimens not recommended by national or international guidelines with resulting suboptimal effectiveness [10]–[14], potentially generating MDR TB. International concern about MDR, extensively and totally drug-resistant strains of TB has also grown recently [15]. The RNTCP started a WHO-recommended DOTS-Plus program for systematic treatment of MDR TB in 2007, though population coverage with DOTS-Plus had only reached 26% in 2011 [16].

In order to predict the likely effectiveness of TB control initiatives currently being considered, it is important to understand the relationship between program effectiveness and the transition from treatment-generated to transmission-generated MDR TB. Reducing transmission-generated cases requires rapid identification and treatment of MDR TB cases to prevent further transmission, while eliminating treatment-generated cases requires improving non-MDR TB cure rates. We first examine the Indian transition from a treatment-generated to a transmission-generated MDR TB epidemic over the previous decade as treatment for TB and MDR TB expanded. Then we project how this transition will change the relative effectiveness of improving non-MDR TB treatment versus shortening the infectious period between MDR TB activation and MDR treatment initiation on MDR TB control.

## Methods

### Overview

We examine the implications of India's MDR TB epidemic for the effectiveness of public health interventions by using a dynamic transmission model of TB calibrated to Indian demography and TB epidemiology. The simulation model represents India's TB epidemic from 1996–2038. The model tracks TB in individuals from the acquisition of latent infection to active pulmonary disease. It follows individuals from birth to death using sex, age, and detailed representations of their TB and MDR TB infection and disease status as well as their case detection, diagnosis, and treatment status and history. These model stratifications are included to allow the model to capture demographically dependent disease dynamics for a complex disease like TB, since mortality, transmission, activation, as well as treatment uptake and effectiveness vary by age and sex in India.

The model was calibrated to match India's TB and MDR TB epidemics between 1996 and 2010, when private-sector treatment continued even as DOTS was scaled up and DOTS-Plus initiated, and it predictions were then compared to multiple epidemiologic and care outcomes to assess simultaneous consistency (see File S1) [17]. We then project incidence, prevalence, and mortality from MDR and non-MDR TB under alternative TB control strategies between 2013 and 2038. We conduct calibration and future projections using a simulated population of 6.5 million people in 1996 that grows to over ten million by 2038, consistent with population growth estimates and projections for India from the United Nations [18]. The large size of the simulated population greatly reduces Monte Carlo noise in the model-estimated outcomes. File S1 details model structure, assumptions, stratifications, calibration, and additional results. Table 1 shows key model inputs.

**Table 1:** Selected model parameters for certain ages and model inputs.

Model Inputs	Value		Source
**Mortality**			
Monthly mortality for 2000–2009	Male	Female	[34]
Age 5	0.0002	0.0002	
Age 25	0.0003	0.0003	
Age 45	0.0008	0.0005	
Age 75	0.0085	0.0071	
Monthly untreated TB mortality for 2000–2009			[35]
Age 5	0.0248	0.0249	
Age 25	0.0275	0.0251	
Age 45	0.0298	0.0257	
Age 75	0.0364	0.0328	
**TB Disease Probabilities**			
Monthly activation probability for latent TB	<2 years ago	>2 years ago	[23]
Age 5	0.0010	0.000432	
Age 25	0.0012	0.000395	
Age 45	0.0010	0.000275	
Age 75	0.0004	0.000267	
Probability of self-cure (all ages)	0		Assumed
Relative infectivity of MDR TB strains (compared to non-MDR TB strains)	1		Assumed
**Sputum Smear Test Characteristics**			
Sensitivity of 3 sputum smear tests for active pulmonary TB	0.60		[28]
Specificity of 3 sputum smear tests for active pulmonary TB	1.00		[28]
**Entering Treatment Probabilities**			
Overall probability of receiving RNTCP treatment if treatment-naive, given that treatment is available	Male	Female	[40] [37]
Age 20	0.1254	0.0477	
Age 40	0.3743	0.1033	
Age 60	0.6000	0.1158	
Overall probability of receiving RNTCP treatment conditional on prior treatment and current treatment availability			[40] [37]
Age 20	0.2821	0.1073	
Age 40	0.6000	0.2325	
Age 60	0.6000	0.2606	
**Treatment**			
Treatment-naive patients			
Probability of death	0.010		[27]
Default probability	Male	Female	[27], [37]
Age 20	0.023	0.018	
Age 40	0.017	0.015	
Age 60	0.022	0.012	
Probability of successful treatment if non-MDR patient completes treatment regimen	0.980		[27]
Previously treated patients			
Probability of death	0.026		[27]
Default probability	Male	Female	[27], [37]
Age 20	0.057	0.043	
Age 40	0.041	0.037	
Age 60	0.054	0.028	
Probability of successful treatment if non-MDR patient completes treatment regimen	0.940		[27]
Probability of testing SS+ at month 4 for non-MDR previously treated patients	0.570		[41]
Probability of developing MDR TB			
If default from treatment	0.242		[27]
If fail treatment	0.187		[27]
Relative infectivity of MDR patient in non-MDR treatment (compared to no treatment)	1		Assumed
Category IV (DOTS-Plus) treatment			
Probability of death	0.017		[42]
Default probability	0.017		[42]
Probability of successful treatment if patient completes treatment regimen	0.738		[42]
Probability treatment suppresses TB to latent infection	0.197		[41]

Please see full list in File S1. Sputum smear positive is abbreviated SS+.

### Dynamics of TB in India

**Figure 1:**
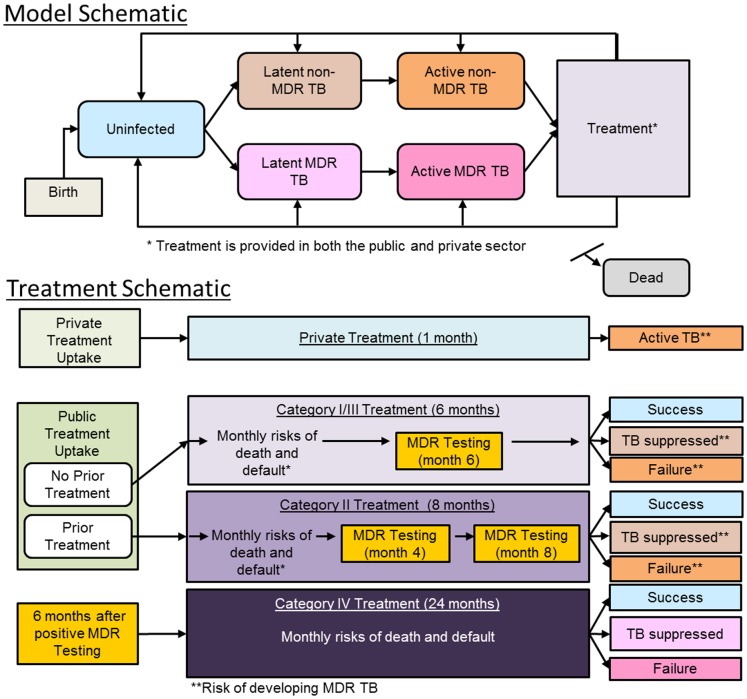
Model and treatment schematic. Model schematic: individuals are born healthy and may subsequently acquire latent TB (non-MDR or MDR) infections through transmission. Individuals who develop active TB disease may subsequently seek treatment. Treatment schematic: individuals with active TB may enter public- or private-sector treatment (see File S1 for details). Individuals in private treatment are not cured but are exposed to a risk of developing MDR TB (see File S1 for details on modeling exposure to the effects of private-sector treatment on MDR generation). Public sector treatment is modeled according to the DOTS protocols: Patients with prior treatment enter category II treatment, and those who test positive for MDR enter MDR treatment (or Category IV treatment/DOTS-Plus) six months after MDR testing if MDR treatment is available. For patients whose non-MDR TB is not cured by treatment, there is a chance that they develop treatment-generated MDR TB.

The model follows individuals through health and treatment states (see Figure 1 top panel). Individuals are uninfected with TB at birth, but may acquire latent non-MDR or MDR infection from individuals with active disease through transmission. Transmission is mediated through an age-stratified mixing process where the likelihood of transmission is determined by respiratory contact rates between different age groups, calculated from empirical age-stratified contact data and India's demographic structure [19]–[22]. Similarly to non-MDR TB, individuals with active MDR TB cases can transmit MDR TB to uninfected individuals. Those with latent infection transition to active disease through an age- and infection-duration stratified process calculated from empirical data on activation in which we assume that latent MDR activates at a similar rate to non-MDR TB [23], [24]. All individuals are exposed to age- and sex- specific background mortality, and those with active TB have an additional disease-specific risk of death.

### TB Treatment in India

Individuals with active disease require diagnosis to begin treatment. Those diagnosed with active disease may undergo treatment under RNTCP protocols or in private-sector clinics (see Figure 1 bottom panel). Treatment duration and effectiveness depend on the individual's diagnosis and true disease status as having either MDR TB or non-MDR TB. Non-MDR disease can be cured with any of the DOTS treatment regimens, while MDR TB can only be cured through DOTS-Plus MDR treatment. We consider non-DOTS treatment in private-sector clinics as generally ineffective against TB [10]–[12], and we explore alternatives to this assumption in the sensitivity analyses.

Individuals with TB symptoms may seek care based on age, sex, and previous treatment status [25], [26]. In order to enter treatment, treatment-seekers must receive accurate TB diagnosis and treatment must be available. Treatment availability depends on RNTCP scale-up: from 1996–2006, the model expands the DOTS TB treatment program coverage consistent with historical data reported by the RNTCP [27], and DOTS-Plus MDR TB treatment is ramped up from 2007–2015 according to expansion plans. We include diagnostic strategies with test characteristics of sputum smear and of rapid MDR diagnostic technologies [28], [29]. For patients with persistently positive sputum smears at 6–12 months after treatment initiation, the presence of MDR TB is assessed via drug sensitivity testing. Depending on the drug sensitivity technology being considered, test results return either the same month or after six months to triage MDR-infected patients to DOTS-Plus [30]. Patient death, default (stopping treatment before completion of treatment regimen), and failure (completing treatment but still having active TB) probabilities for each DOTS treatment category depend on sex and age and were derived from RNTCP-reported statistics. Individuals treated for non-MDR TB who default from or fail treatment may develop treatment-generated MDR TB. As a simplification, the model assesses MDR TB status at the time of default or failure, although the actual biological process of resistance selection is continuous over the treatment period. Those who are cured via treatment may become infected again through transmission and those who exit from treatment with latent TB may later reactivate. We assumed that patients with MDR TB on inappropriate treatment (DOTS instead of DOTS-Plus) are not cured and have the same infectiousness as without any treatment.

Private clinics may be an important contributor to India's MDR epidemic and are included in the model (see Figure 1 bottom panel). Individuals with active TB who have not been treated in the RNTCP system may seek private clinic care. We match empirical estimates of treatment-seeking rates in private clinics prior to entering the RNTCP system [14], [31]. We conservatively assume that the MDR acquisition rate from private sector treatment is similar to that of defaulting from public sector treatment and acquiring MDR TB, and we explore this assumption in the sensitivity analyses.

### Scenarios: Changes in non-MDR Treatment Quality and Treatment Timing

In order to explore how the changing MDR epidemic could alter the effectiveness of control efforts, we examine the effect of two policies that target treatment-generated and transmission-generated MDR. One approach is to further improve non-MDR treatment. Improving non-MDR treatment directly reduces the number of treatment-generated MDR cases by reducing cases of incomplete or ineffective treatment that may lead to development of MDR TB strains. We examine the effect of improving non-MDR default, mortality, and success rates to match those of the best-performing Indian state RNTCP program in 2010 (see Table 2). These targets might be achieved through better clinical monitoring and patient outreach and education to quickly identify and remedy medical complications or suboptimal patient response.

**Table 2:** Analysis Scenarios.

	Base Case	Intervention: Improving non-MDR treatment quality	Intervention: Improving rapidity of MDR diagnosis
**For 30-year old male, Non-MDR Treatment Probability of** * **:**			
CAT I/III**			
Death (monthly)	0.010	0.003	Same as base case
Default (monthly), conditional on alive	0.021	0.005	Same as base case
Failure, conditional on completion	0.980	0.990	Same as base case
CAT II			
Death (monthly)	0.026	0.015	Same as base case
Default (monthly), conditional on alive	0.052	0.007	Same as base case
Failure, conditional on completion	0.940	0.972	Same as base case
**Timing of first drug sensitivity testing**			
CAT I/III	Month 4	Month 4	At initial patient assessment
CAT II	Month 8	Month 8	At initial patient assessment
**Duration before test results return**	6 months	6 months	1 month

*Treatment death, default, and failure rates vary by age and sex. Probabilities for 30 year old males are used here as an example. Default probabilities are conditional on being alive, and failure probabilities are conditional on being alive and completing treatment. Probabilities for other ages and sexes for base case and improving non-MDR treatment quality (best state outcomes) are given in Table 1 and File S1 table S5.8.

**Treatment categories refer to DOTS treatment category I/III and category II, as explained in the text in section Methods: TB Treatment.

A second approach is to target transmission-generated MDR by decreasing the time between activation of MDR TB and effective treatment by improving the rapidity of MDR diagnosis. Differentiating MDR from non-MDR TB and placing MDR patients on appropriate treatment in the first month after they begin directly reduces transmission-generated MDR by shortening their infectious period prior to receiving appropriate treatment. Currently it can take a patient almost 12 months from entry into non-MDR treatment to be identified with MDR TB and placed on MDR TB treatment, as patients are not tested for MDR immediately after entering treatment and long test turn-around times delay appropriate treatment after diagnosis [36]. To accomplish this, guidelines could be changed to recommend immediate MDR testing upon entry to treatment, and reductions in test turn-around periods might be realized by using more rapid drug sensitivity testing technologies, reducing administrative delays in test processing, and maintaining efficient supply and information networks between patient facilities and laboratories performing drug sensitivity testing.

We examine the effectiveness of these two control strategies and explore how a transition from a treatment- to transmission-generated MDR TB epidemic alters their effectiveness in reducing the prevalence of infectious MDR TB (i.e., the prevalence of MDR TB cases not on effective treatment). We consider hypothetical scenarios in which these approaches are implemented in 1997, 2007, 2017, and 2027. The 1997 and 2007 scenarios benchmark what might have happened if these approaches had been implemented when MDR TB levels in India were relatively low. The 2017 and 2027 scenarios illustrate the effect of prompt versus delayed implementation of such measures. We compare outcomes under these scenarios to the base case of continuing at current quality levels with MDR treatment programs scaling up as scheduled. We also consider combinations of the two strategies to assess their interaction in the presence of MDR TB epidemic transitions.

Given current interest in public-private TB treatment efforts to improve outcomes in private-sector clinics [32], [33], we also use the model to examine the role of private clinic treatment in India's MDR TB epidemic. We therefore model the prevalence and mortality caused by MDR TB in the hypothetical scenario where, instead of DOTS having been implemented, private sector treatment expanded to cover 50% of the population currently covered by DOTS.

### Model Calibration

Because no studies existed to provide direct measures for several model inputs in the Indian context, we calibrated the effective transmission risk (the probability of TB transmission given contact between a susceptible and infectious individual), the average rate of TB activation (the average rate at which latent infection transitions to active disease), and the average treatment take-up rate to match model outputs to empirical data on TB prevalence, incidence, and RNTCP patient demographics (see File S1, specifically Table S2). We chose the set of calibration inputs that maximized the number of annual modeled outputs that fell within uncertainty bounds of the empirical data provided by the literature (for all three types of output measures). We then performed a face validation of the model by comparing model outputs on demographic and disease measures which were not calibrated to other empirical data from the literature (see File S1, specifically sections Calibration and Face Validation). The calibrated model produced outputs simultaneously consistent with many demographic and disease measures [34], such as prevalence and incidence of TB over time [1], [35], WHO estimates of MDR prevalence [36], and RNTCP-reported overall and age- and sex-specific treatment utilization levels [8], [13], [27], [37]. Additionally, given uncertainty in calibration targets and necessary assumptions, we consider numerous sensitivity and scenario analyses to gauge how much particular assumptions influence model predictions.

### Sensitivity Analyses

Effective MDR TB control depends in part on MDR treatment availability, but MDR treatment has not yet reached nationwide coverage [16]. We explored scenarios in which the planned expansion of India's MDR treatment program is delayed or halted, and examined the impact on the effectiveness of our disease control policy scenarios. There is also uncertainty surrounding the average transmission fitness of MDR TB strains relative to non-MDR TB strains, and we explore how our results change if MDR TB is less transmissible than drug-sensitive strains. In addition, we also examine the effect of attenuating our rapid diagnosis policy, varying non-MDR cure rates in private clinics, and varying the probability of acquiring MDR TB in private clinics. Finally, there is uncertainty about how quickly latent infections activate; therefore we examine how varying rates of activation within bounds cited in the literature impact the results of the analysis (details in File S1).

## Results

### Calibration to TB Epidemic Trends

**Figure 2:**
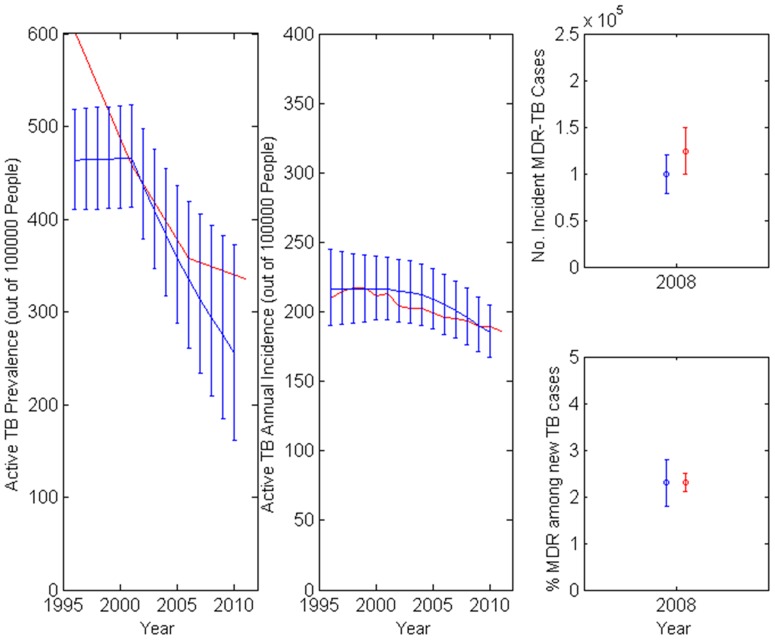
Calibration results: comparison of WHO estimates of TB prevalence and incidence in India (blue lines) to modeled outcomes (red lines). The simulation model's output matches WHO reports on Indian TB prevalence and incidence, fitting time trends for non-MDR TB in 1996–2012 and WHO estimates of MDR TB in 2008.

Calibrated model outputs for India's annual overall TB incidence, MDR TB incidence, and TB prevalence between 1996 and 2012 simultaneously matched empirical estimates (additional calibration results in File S1). Consistent with Indian data, the model shows that increasing DOTS treatment coverage reduced prevalence of latent and active non-MDR TB (Figure 2). We find that active TB prevalence has declined by 290 per 100,000 between 1996 and 2010 – within the uncertainty bounds of WHO estimates [1].

### Base Case Projections for non-MDR and MDR TB Trends

**Figure 3:**
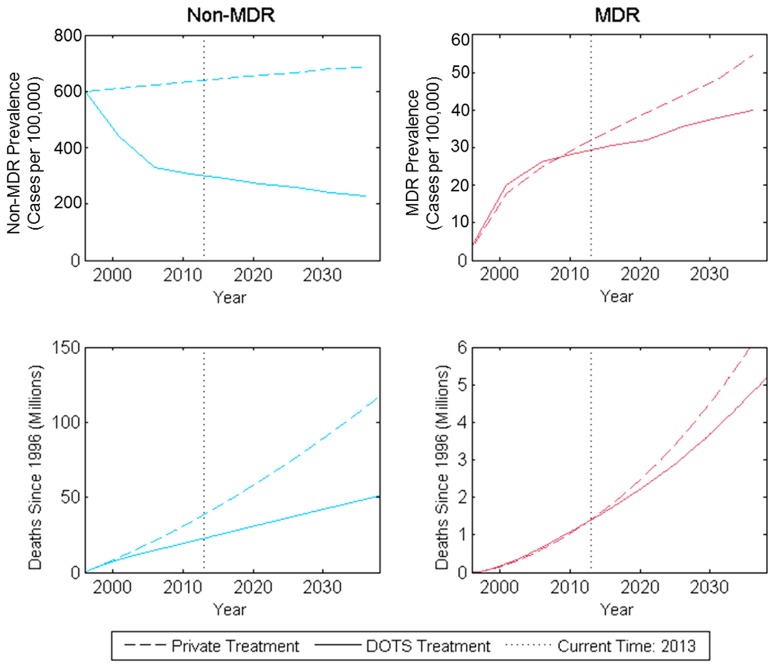
Projected prevalence and mortality from non-MDR and MDR TB in India with public DOTS treatment programs and counterfactual private treatment expansion in the absence of public treatment. Figure shows model estimations and projections of disease prevalence and deaths after 1996, when public nationwide TB treatment in India began. Private treatment curves (dashed lines) represent outcomes in a scenario where DOTS was never implemented and private clinic population coverage increased to half of the level that DOTS currently covers. Solid lines represent disease prevalence and deaths given observed public treatment levels in India and assume public TB treatment will continue at current levels.

Continuing non-MDR treatment at current coverage and treatment success rates is projected to provide substantial health benefits for individuals with non-MDR TB in the future relative to a scenario with no effective TB treatment (see Figure 3). DOTS treatment decreases the prevalence of active non-MDR TB from 0.29% to 0.21% of the total population in 2038 (compared to 0.68% without treatment). Continuing treatment until 2038 is projected to prevent 295 million latent TB infections. Additionally, by averting 24 million active TB cases in this period and improving the prognosis of the remaining active TB cases, treatment is also projected to avert 48 million TB-related deaths.

Current levels of TB treatment and control in the public sector have led to lower levels of MDR TB prevalence than if TB treatment had been provided exclusively by private-sector clinics that do not follow effective TB treatment protocols. If DOTS had never been implemented and instead the private sector had expanded to cover half of the population, MDR TB prevalence would be approximately 33% larger in 2038 – rising from 32 per 100,000 in 2013 to 56 per 100,000 in 2038 (see Figure 3).

However, even with DOTS and the planned scale-up of DOTS-Plus in the public sector, MDR TB is projected to grow through 2038 if additional measures are not taken (see Figure 3). Active MDR TB in the overall population will increase from 29 per 100,000 in 2013 to 42 per 100,000 in 2038 (an increase of 13 per 100,000). Under the scenarios we consider, this estimate ranges from 19–35 cases per 100,000 in 2013 and the increase ranges from 7–15 per 100,000 (see Figure S20 in File S1). Notably, the MDR prevalence trend continues to rise in all scenarios considered. Over this period, our base case analysis indicates that India will experience 5.2 million incident cases of active MDR TB, causing 3.9 million deaths, even as effective MDR TB treatment becomes more widely available.

**Figure 4:**
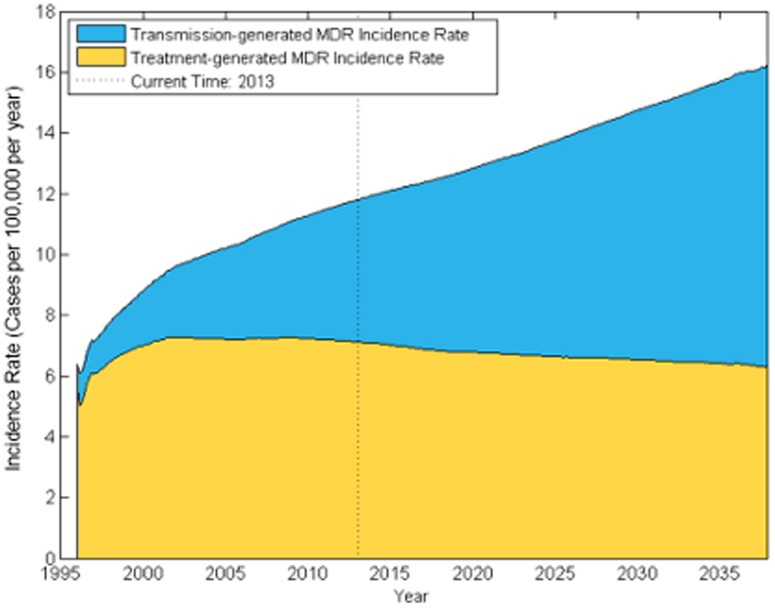
Projected incidence rate of treatment-generated and transmission-generated MDR TB. The fraction of the Indian population with incident MDR TB disease is shown over time. The blue region represents the fraction of the population with incident transmission-generated MDR TB, while the yellow denotes the fraction with treatment-generated MDR TB.

The source of incident MDR TB cases is changing. The model estimates of transmission- and treatment-generated MDR TB levels generally fell within confidence intervals for the WHO estimates of the number of transmission-generated MDR cases in 2008 – 52,000 (95% CI 47,000–56,000); WHO: 55,000 (95% CI 40,000–74,000) – and the number of incident treatment-acquired MDR cases: 73,000 (95% CI 52,000–94,000); WHO 43,000 (95% CI 33,000–56,000) [36]. We find that by 2013, the shift towards transmission as a major source of incident MDR is well underway, with 40% of new MDR cases transmission-generated. Projecting into the future, primary MDR TB transmission will be responsible for a growing proportion of MDR TB cases relative to MDR TB cases generated from ineffective, discontinued or unsuccessful non-MDR treatment (Figure 4). In the various scenarios we consider, transmission-generated MDR TB ranges from 31% to 41% of new MDR cases in 2013, with the lower extreme from a scenario where the transmission fitness of MDR TB is assumed to be 70% of non-MDR TB (see File S1 for details). The model projects that transmission-generated MDR will continue to rise even if India's MDR treatment program expands to nationwide availability by 2015, highlighting the need for additional MDR TB control efforts. MDR TB prevalence rises in all of our sensitivity scenarios, summarized by Figure S20 in File S1. However, our estimates of increasing MDR-TB incidence over time depend on our assumptions regarding age-specific treatment-seeking and mixing behavior; assuming no age-dependent treatment seeking or mixing results in MDR incidence and prevalence projections that are much flatter over time than in the main analysis.

### Implications for MDR TB Control Efforts

**Figure 5:**
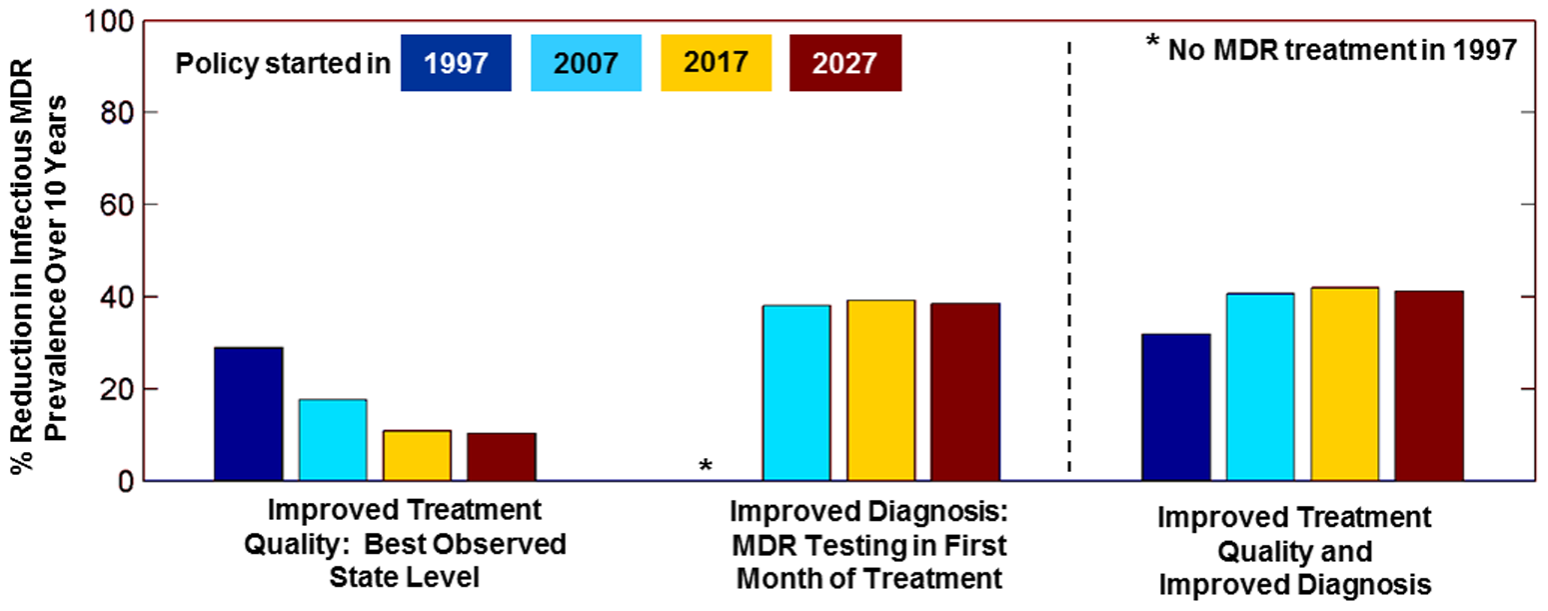
Reduction in MDR TB prevalence with improvements in treatment of non-MDR TB and diagnosis of MDR TB. The figure shows the average percentage reduction in infectious MDR prevalence over ten years after the improvements begin (either in 1997, 2007, 2017, or 2027).

The MDR control benefits to India from improving non-MDR TB treatment are shrinking, though there are still important direct benefits for reducing non-MDR TB prevalence and incidence. Because India's MDR TB epidemic is expected to continue transitioning from a treatment-generated towards a transmission-generated epidemic, the impact on MDR TB of improving non-MDR TB treatment declines over time. In contrast, the impact of improving the rapidity of MDR TB diagnosis remains constant (see Table 3 and Figure 5). For example, improving non-MDR TB treatment across India to best-observed levels in 2007 would have resulted in a 17% reduction in the prevalence of infectious MDR TB cases over the following 10 years; in contrast, doing so by 2017 results in a 10.8% reduction and a 10.3% reduction by 2027. Implementing more rapid MDR diagnosis in 2017 results in a 39% reduction in infectious MDR TB prevalence, and effectiveness remains stable even if the policy is implemented later. When quality improvements and improved MDR TB diagnosis are implemented together, the percent reduction in infectious MDR prevalence is larger than either alone for all policy start times, indicating that there may be benefits to combining MDR control strategies (Figure 5). Notably, across all scenarios considered, the model projects that none of the policies evaluated can eradicate MDR TB in the next 25 years (see Figure S21 and S22 in File S1).

**Table 3:** Results summary.

	Base Case	Intervention: Improving non-MDR treatment quality	Intervention: Improving rapidity of MDR diagnosis
**Average 10-year Infectious MDR TB Prevalence starting after intervention completes in (cases out of 100,000):**			
1997	22.4	16.0	22.5
2007	22.5	18.5	14.0
2017	25.4	22.7	15.4
2027	30.2	27.1	18.6
**Percentage reduction in infectious MDR TB prevalence (compared to analogous base-case time period):**			
1997	NA	28.9%	NA (No DOTS-Plus)
2007	NA	17.6%	38.0%
2017	NA	10.8%	39.2%
2027	NA	10.3%	38.5%

Percentage reduction in infectious MDR TB prevalence also shown graphically in Figure 5.

Failure to increase population coverage of MDR treatment programs beyond the current level of 26% strongly influences the MDR control benefits achieved through improved MDR diagnosis. We assessed the benefits from improved MDR diagnosis if MDR treatment program coverage did not expand as currently planned. If MDR treatment coverage remained at 2011 levels, the benefits from improving MDR testing would decline by 7% relative to the scenario where MDR treatment fully scaled up by 2015 (from 39% to 32% reduction in MDR TB). Longer term effects on various MDR control strategies remain similar to those in the main analyses even if MDR treatment scale-up is slower than expected (see sensitivity analyses in File S1).

Because of uncertainties about patterns of TB care and TB disease natural history that have potentially important implications for MDR TB control, we examined how alternative assumptions impact the effectiveness of the policies we consider. Specifically, we examined assumptions about delaying MDR TB treatment initiation even after rapid MDR diagnosis (after two months vs. within the first month in the base case); non-MDR TB cure rates in private clinics (22% vs. 0% in the base case); rates of MDR TB generation from treatment in private clinics (0.03x–1.70x of the base case rate); the rate and heterogeneity of latent non-MDR and MDR TB activation (see sensitivity analyses in File S1); and transmission fitness of MDR TB relative to non-MDR TB (70% vs. 100% in the base case) [38]. Across these scenarios, our results remain robust with non-MDR TB treatment quality improvement having less of an effect on MDR control if implementation is delayed while rolling out rapid MDR diagnosis maintains its effectiveness even if implementation is delayed. See File S1 for further details.

## Discussion

The rise of MDR TB presents serious challenges to TB control. We provide results from a detailed TB epidemic model that illustrate the implications of India's transition from a treatment-generated MDR TB epidemic towards one that is dominated by transmission-generated disease. This shift has important implications for disease control policies, as programs that target treatment-generated MDR TB are predicted to become less effective. As transmission-generated MDR TB becomes a larger driver of the MDR TB epidemic in India, rapid and accurate MDR TB diagnosis and treatment will become increasingly important for reducing MDR TB cases compared to non-MDR TB treatment improvement.

We show that transmission is likely to play an increasingly important, direct role in driving India's MDR TB epidemic. A reservoir of prevalent latent MDR TB infections has been accumulating, originally infected from incident treatment-generated active MDR TB cases. The activation of these latent infections contributes to the growth of incident active MDR TB and, without rapid identification and treatment, can generate a self-sustaining MDR TB epidemic. As a result, rapid identification and treatment of incident MDR TB is increasingly effective as the transmission-generated epidemic grows in importance, though the magnitude of this increase depends on making effective MDR treatment widely available. Correspondingly, our findings show that the window of time available for controlling the growth of MDR TB by improving non-MDR treatment is closing. This has public health implications given the duration, toxicities, costs, and complexities associated with MDR TB treatment. Our findings suggest that successful efforts to address MDR TB in India will require understanding the source of new infections and tailoring disease control measures based on the relative contribution of treatment-generated versus transmission-generated infections to the MDR epidemic.

### Study Limitations

The simulation model excludes extra-pulmonary TB because its mode of transmission differs significantly from pulmonary disease, and pulmonary TB contributes over 80% of the TB cases in India. The model does not explicitly account for the effect of co-infections such as HIV or for risk factors such as malnutrition, which are implicitly incorporated through the use of India-specific data sources and calibration. The prevalence of HIV among individuals with active TB in India is relatively low compared to other high-prevalence TB countries, such as South Africa, and may not have substantial influence on India's overall TB epidemic dynamics. We assume the probability of self-cure for both non-MDR and MDR TB to zero and note that there are few data with which to inform self-cure rates in settings like India. To the extent that self-cure does occur in this context, the model may still implicitly incorporate this effect via its activation and transmission rates as both are calibrated to match WHO reports on TB incidence rates through the 1990s. Explicit estimation and incorporation of self-cure rates is left to future work.

The model also does not explicitly account for mixed-strain infections, where individuals may be simultaneously infected with both non-MDR and MDR TB; we assume non-MDR TB infection is protective and individuals cannot be additionally infected with MDR TB. As latent non-MDR TB infection prevalence in India is high, if the likelihood mixed-strain infection were sufficiently large, our model would underestimate the rate of MDR TB growth, though it would also likely underestimate the effectiveness of the interventions we considered. The model similarly omits explicit consideration of XDR TB from the analysis as little is known about XDR selection and transmission dynamics in the context of India. The incorporation of extra-pulmonary TB, HIV, and other risk factors is left to future work.

Much uncertainty remains around MDR measures (such as average patient response rates in non-MDR treatment, self-cure rates, relative infectivity when on non-MDR treatment and without, etc.). These can substantially change the growth rate of MDR TB prevalence and incidence, though we note that sensitivity analyses around these parameters show that the model results remain robust regarding decreasing effectiveness of improved treatment quality and constant effectiveness of improved MDR diagnosis policies. We therefore leave the detailed exploration of these MDR measures to future work.

The model does incorporate age, sex, and behavioral risk factors, such as differences in treatment-seeking behavior. We include age- and sex-stratifications in the model to more accurately capture mortality, disease transmission and activation, and treatment uptake and outcomes, which differ by age and sex and generate dynamics important for predicting disease outcomes. However, these stratifications may also introduce additional uncertainties as the number of model inputs increase, and simpler models may also be more transparent. We believe that inclusion of these stratifications is justified because the model must match empirically measured outcomes that depend on age and sex, which is much harder to do without age- and sex-stratification.

The purpose of the study was to characterize how shifts in India's MDR TB epidemic over time from treatment-generated cases to transmission-generated cases impact the effectiveness of general classes of control measures aimed at MDR TB. In general, these policies may target MDR generation in treatment (such as increasing non-MDR treatment quality in the RNTCP, as discussed in this paper, or other methods, such as reducing private clinic use through referral programs, etc.) or may try to limit direct MDR transmission (by reducing time to effective MDR treatment, as discussed in this paper, or potentially through other measures such as limiting transmissible contacts, etc.). While we illustrate the implications of this transition on the effectiveness of two example policies, we have not specified the means by which DOTS treatment is improved and cannot make specific recommendations about which policies should be implemented as our study did not include all potential policies, assess feasibility, or consider costs. Demonstration studies considering rapid MDR diagnostics in India are underway [39] and will likely contribute important data to refine model estimates and perform policy analyses in future work. Even so, our current results on decreasing efficacy of non-MDR TB treatment-improvement policies to control MDR TB offer important cautionary information for near-term planning.

Our analysis focuses on MDR TB in the context of India's general TB epidemic, and identifies how epidemiological trends may alter the effectiveness of control of non-MDR TB. We illustrate and quantify the reductions in non-MDR TB burden with the growth in India's treatment programs. We estimate that by 2038, TB treatment programs in India will contribute to a substantial decline in TB incidence, averting 48 million TB-related deaths through effective TB case management. However, the incidence of MDR TB is growing. Taken together, the projected declines in non-MDR TB and increases in MDR TB further emphasize the growing role of drug resistant disease and the need to critically consider MDR control measures.

## Supporting Information

File S1 **Supporting Information.**
(DOCX)
