# Supplementary material for: Disease Control Implications of India's Changing Multi-Drug Resistant Tuberculosis Epidemic
Source: PLoS One. 2014 Mar 7;9(3):e89822. doi: 10.1371/journal.pone.0089822 (PMC3946521; doi:10.1371/journal.pone.0089822)
Supplement: File S1 — Supporting Information. (DOCX) [file pone.0089822.s001.docx]

**Appendix: Supporting Information for**

“Disease Control Implications of India’s Changing Multi-Drug Resistant Tuberculosis Epidemic”

Sze-chuan Suen, Eran Bendavid, Jeremy D. Goldhaber-Fiebert

Table of Contents

Appendix Section 1: Model Description 2

Model Overview3

Natural History3

Birth Rates3

Death Rates3

TB4

TB Transmission 4

TB Activation6

TB Treatment6

TB Treatment: Public Sector TB Ramp Up6

TB Treatment: Diagnosis7

TB Treatment: Public Sector Treatment7

TB Treatment: Private Sector Treatment10

Calibration10

Analysis Scenarios 11

Treatment-Generated and Transmission Generated MDR TB12

Appendix Section 2: Internal and Face Validation14

Appendix Section 3: Sensitivity Analysis16

Effect of Missing MDR Treatment Coverage Targets16

Effect of Differential MDR Transmission Fitness17

Effect of Delay in MDR TB Treatment Even After Rapid MDR TB Diagnosis 17

Effect of Private Clinics Achieving Cure for Non-MDR TB for a Proportion of Patients 17

Effect of Variation in Private Clinic MDR TB Generation Rate18

Infectious MDR TB Prevalence Over Time19

Effect of Alternative Activation Parameters21

Model Inputs Table23

S5.1: Initial Cohort Parameters23

S5.2: Population Growth25

S5.3: Mortality25

S5.4: TB Activation28

S5.5: Sputum Smear Test Characteristics29

S5.6: MDR Testing Parameters29

S5.7: Treatment Uptake Parameters29

S5.8: Treatment Parameters30

S5.9: Transmission Parameters32

References33

**Appendix Section 1: Model Description**

We developed a dynamic transmission, microsimulation model of non-MDR and MDR TB in India using MATLAB® (2011b, The MathWorks, Natick, MA). Our model tracks individuals matched to the population of India in terms of age, sex, and TB status. The model includes a detailed simulation of TB transmission, infection and disease as well as case-detection, diagnosis, and treatment. The model was calibrated to statistics on India’s TB epidemic between 1996 and 2012 as DOTS therapy was scaled up in an environment in which non-standard treatment continued to be delivered through private health clinics. We then used the calibrated model to consider the future epidemiology of non-MDR and MDR TB and resulting disease burden in India over a 25 year time horizon (2013-2038). The model has the capability of examining the relative effectiveness and outcomes of TB control policies and technologies in the presence of India’s changing TB epidemiology.

*Model Overview*

*
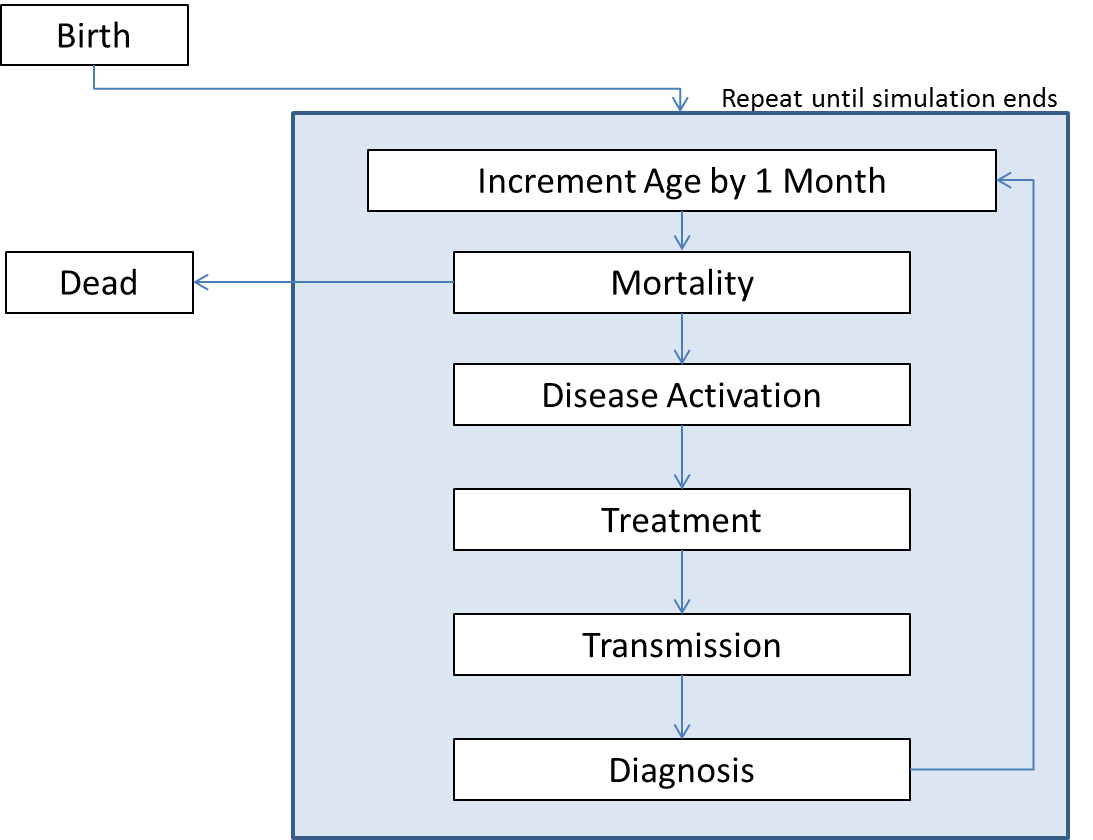
*The model proceeds in one-month time steps. Each month, the model tracks and updates the following events: births; deaths; transmission of non-MDR or MDR infections; care seeking and treatment in the private healthcare sector; initiation, default from, and completion of DOTS or DOTS-plus treatment in the public sector; and the development of MDR TB due to selection pressures from treatment that does not result in cure whether due to inappropriate regimens, insufficient duration, or other factors. The overview of the various processes modeled in the simulation is shown below (Figure S1).

Figure S2: Detailed Model Schematic: Overview

Figure S1: Detailed Model Schematic: Overview

Prior to making future projections, the model is run in several stages to ensure high fidelity with India’s TB epidemiology. First, to produce a stable population in terms of demographics (age structure, sex balance, life expectancies, etc.) and TB (latent and active TB prevalence and incidence) that match 1996 levels in India, we employ a procedure termed “model burn-in.” The simulation begins with an initial population of 480,000 individuals (See Table S5.1 for starting population values in the model) and then runs for 130 years (i.e., 1,560 months, the “burn-in” period). During this period, the population is subject to the simulation’s monthly probabilities of birth, disease, and death. We hold disease and demographic transition parameters fixed during this period to arrive at a steady state. Next, the model is calibrated to a variety of Indian demographic and TB epidemiologic data as it is run from 1996-2012 – the period when India’s public TB treatment program, referred to as DOTS, was scaled up. The model similarly scales up DOTS in this period. Finally, the calibrated model is run for 25 years to perform analyses and make projections (years 2013-2038). Population growth in the model is consistent with that observed in India. We conduct calibration and future projections using a simulated population of 6.5 million people in 1996 that grows to ten million by 2038, consistent with population growth estimates and projections given by the United Nations [1]. For computational tractability reasons, the model’s simulated population consists of a smaller number of individuals than are in the actual Indian population. We scale the numbers in the model to the total Indian population size to consider impacts on absolute disease burden (thus, proportions of the modeled population are multiplied by the corresponding size of the actual Indian population in a given year).

*Natural History*

The simulated population is stratified by characteristics including age, sex, TB disease status (latent or active), and TB type (non-MDR or MDR). Extra-pulmonary TB is not considered in this analysis. Transition probabilities and other model parameters were stratified by as many characteristics as the literature allowed (see Model Input tables at the end of the SI for particular stratifications and values). When it was not possible to stratify a particular parameter by a given characteristic, equality across that characteristic (e.g., males and females) for that parameter was assumed. The sections below show how data from the literature were used to compute model inputs and, where appropriate, how they were stratified by these various characteristics.

In the simulation model, individuals are described by a set of characteristics that may be fixed at birth (e.g., sex), progress deterministically (e.g., age), or be subject to chance events (e.g., infection with non-MDR TB). The combination of an individual’s current characteristics and past experiences may influence subsequent event risks. Each individual has the following characteristics:

- Sex (male/female)
- Age (year and month)
- Health status (healthy/latent non-MDR infection/latent MDR infection/active non-MDR disease/active MDR disease/dead)
- Date when infected with TB (null if never infected; infection date if ever infected)
- Current treatment status (none/ CAT I/III / CATII/DOTS-plus)
- Number of months in treatment if currently in treatment
- Date when individual began MDR testing (null if individual has never initiated MDR testing, completed MDR testing and tested negative, or completed DOTS-plus / MDR testing initialization date otherwise)


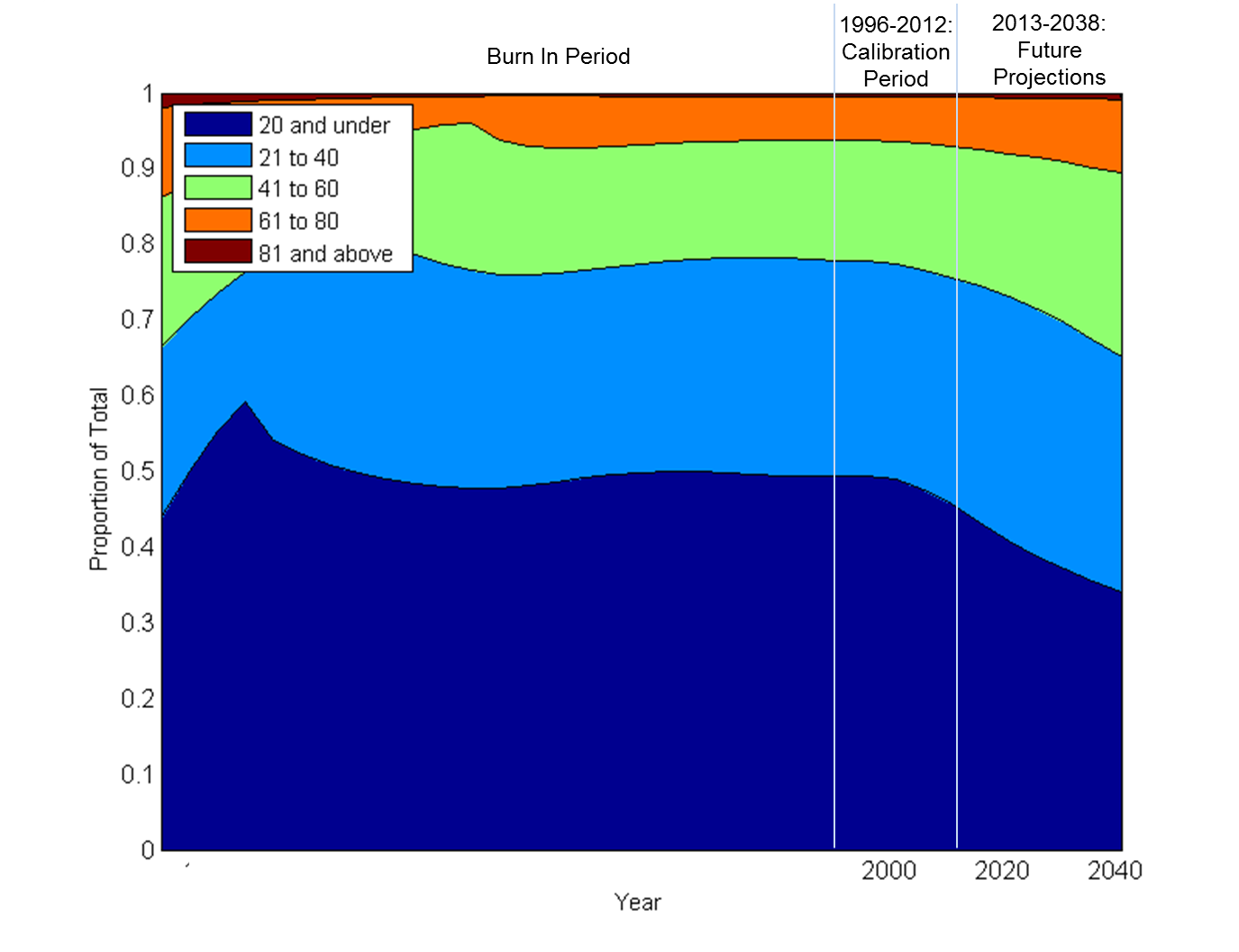


Figure S2: Model Population Age Structure Over Simulation Time

- Past treatment history (Never had treatment/Had treatment and completed/Had treatment and defaulted)
- Whether individual is willing to seek treatment in DOTS if he develops active TB

*Birth rates*

Individuals are born into the model based on historic population growth and United Nations projections of future population growth using the “medium” growth trends [1]. Population growth percentages were applied to the model population to generate the number of births in each month. Along with births, the population age structure is indirectly determined by the current population size along with age and sex-specific mortality rates from WHO life tables (see Death rates below). The resulting age structure in the model stabilizes during the “burn in” period, and shows population aging thereafter, especially over 2013-2038 (see Figure S2).

*Death rates*

Every month individuals are exposed to the risk of death (see Figure S3). Mortality rates in the model are age-, sex-, and TB disease-specific. Age- and sex- specific Indian mortality data was derived from the WHO 1990, 2000, 2009 Indian life tables [2]. The 1990 mortality rates were used for the burn in period and simulation years prior to 2000; the 2000 rates for years prior to 2009; and the 2009 rates for years after this including the future projections through 2038. These age- and sex-specific derived mortality rates were further modified by excess risks of death for untreated and effectively treated TB disease (see section below on TB Treatment). Latent infection was not associated with increased mortality risk. Untreated TB mortality rates were derived from the literature.


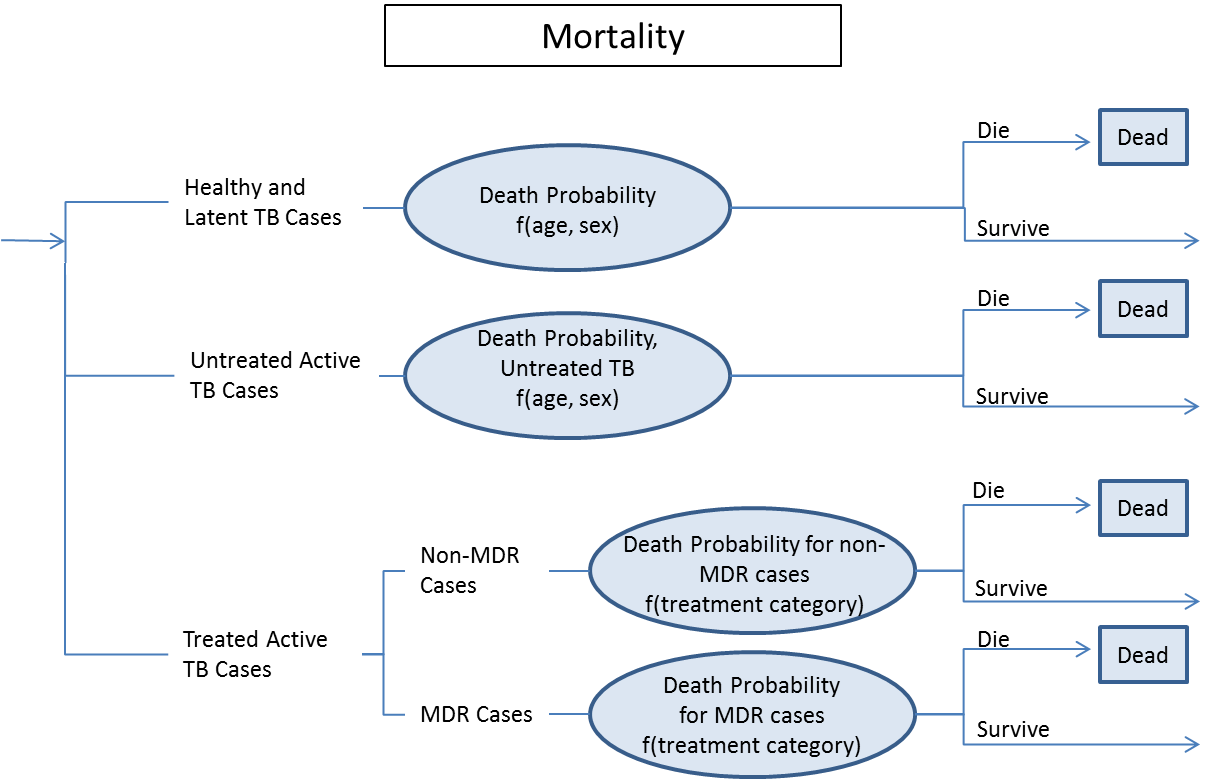


Figure S3: Detailed Model Schematic: Mortality

*TB*

Individuals can be healthy (uninfected), have latent TB infection (non-MDR or MDR), or have active TB disease (non-MDR or MDR). We assume that the risks for individuals with latent TB infections are identical to otherwise similar healthy individuals with the exception that their TB infection may activate at a rate dependent on their age and duration with latent infection (see Figure S4). MDR TB differs from non-MDR TB in that it cannot be cured by non-MDR TB treatment but rather only by MDR TB treatment. The prevalence of latent and active TB at the time of model initiation is based on estimates from the literature [3]. After the model is burned in for 130 years, we use estimates from the WHO MDR/XDR 2004-2007 Report to convert a proportion of latent and active TB cases to MDR TB to simulate the latent disease profile of India in 1996. After calibration, model estimates of latent and active MDR match those estimated by Dye et al. [3] for 1999, time trends in disease incidence and prevalence [4], and a variety of demographic and treatment measures (see appendix section “Calibration” and “Appendix Section 2: Internal and Face Validation”)


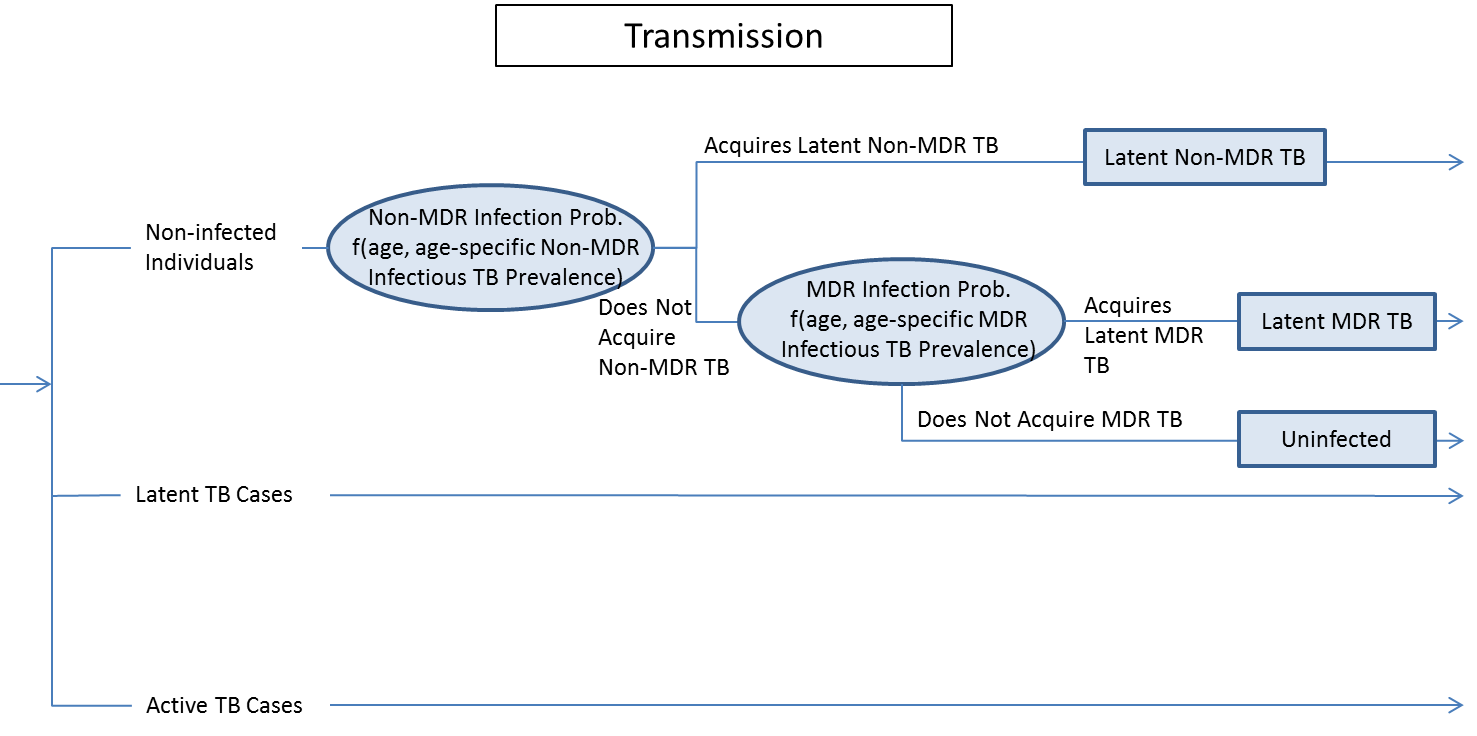


Figure S5: Detailed Model Schematic: Transmission


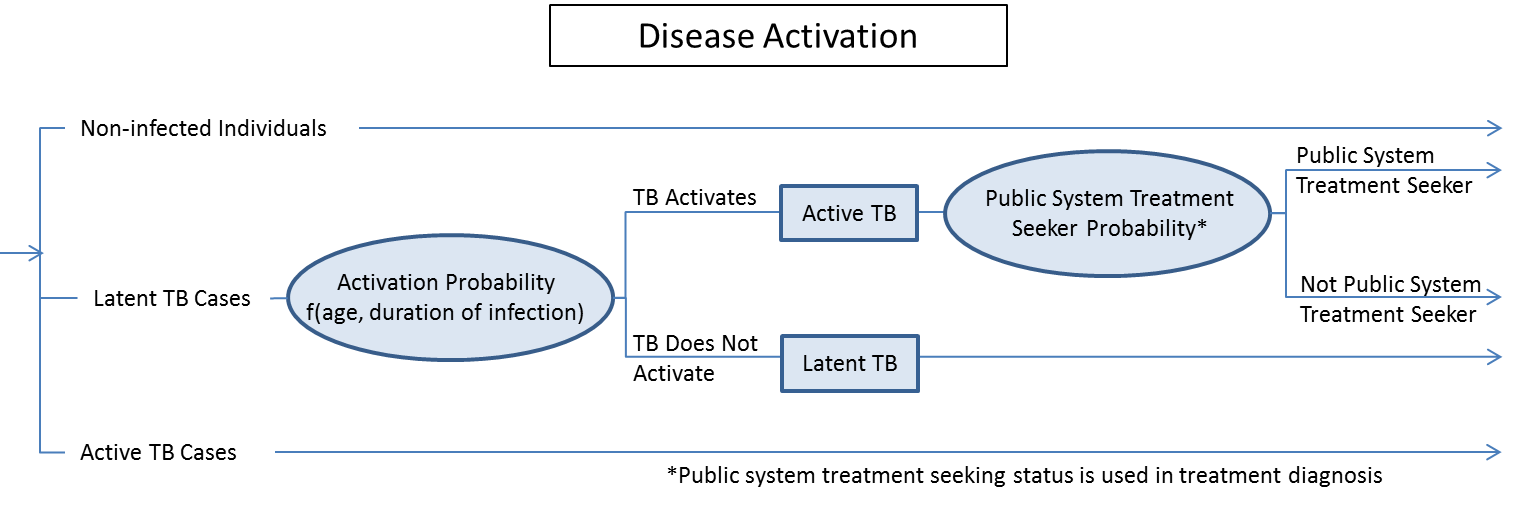


Figure S4: Detailed Model Schematic: Activation

*TB Transmission*

Individuals with untreated or ineffectively treated active TB can infect susceptible individuals --healthy/uninfected individuals (see Figure S5). Those with latent infections cannot be re-infected with another strain; rather, the rate of activation implicitly captures the possibility of activation after re-infection. As a simplification, we do not allow the possibility that individuals with latent non-MDR TB will be infected with MDR TB during latency. Transmission can occur each month. Non-MDR TB and MDR TB infections are transmitted separately (i.e., non-MDR cases can only transmit non-MDR TB, MDR TB cases transmit MDR TB).

We use a who-mixes-with-whom (WMW) age-stratified transmission approach [5], where individuals in each age group have different contact rates with individuals of various age groups, leading to differential age-specific probabilities of TB transmission. As no such matrix has yet been estimated for India directly, the original WMW contact data is taken from Mossong [6], who conducted a study on physical contacts in Great Britain (GB) as well as in other European countries. We modified the contact matrix estimated for Great Britain to reflect differences in Indian household composition and other social differences that may alter the age-structure of contact patterns. We compared these changes to who-mixes-whom-matrices estimated from a smaller study in Vietnam whose social mixing patterns may be closer to India [7]. Like the one for Vietnam, our contact matrix shows strong same-age as well as intergenerational mixing patterns (off-diagonals) -- for instance, this would mean that 30 year olds mix more with other 30 year olds (their peers) and 5-15 year olds (their children) than they do with 20 year olds. It also has families having children at relatively younger ages (distance between main diagonal and off diagonal), and the matrix is also asymmetric to reflect the lower infectivity of children compared to adults [8,9]. Figure S6 shows the original contact matrix and the one adapted for India.

Modified WMW for India

Original WMW from GB

Figure S6: Original and Modified WMW Matrices. The colors represent the number of daily respiratory contacts between age groups. The panel on the left is a visual representation of the original contact matrix from Great Britain (GB) [6] while the right reflects the matrix adapted to the Indian context. Changes made included: a) reducing same-age mixing rates for children younger than 5 given lower levels of out-of-home daycare use; b) increasing contact rates in the minor diagonals roughly ages 17 and 34 years from the same-age mixing diagonal, representing stronger intergenerational mixing patterns for children in India versus in Great Britain [10], consistent with mixing patterns seen in other developing countries like Vietnam [7]; c) The lower minor diagonals were additionally strengthened to incorporate the higher probability of transmission from parent/grandparent to child than vice versa [8,9]. Changes were made while preserving the row sums of the original matrix in order to maintain relative overall levels of contacts.

In the simulation model, the contact matrix adapted to India is used to compute age-specific transmission probabilities. As a simplification, we assume random mixing between sexes within contacts in each age-specific set of contacts. The contact matrix is instrumental in determining the expected number of new infections of type *k* (i.e., non-MDR or MDR TB) in one month in age group *j* generated by infected individuals in age group *i*, if age group *j* were completely susceptible (i.e., not latently infected with any type of TB or with active TB disease), as given by the formula:

*a_ik_ * n_ijk_ * p*

where:

- *i*, *j* index age group of infectious individuals and contacts, respectively
- *k* indexes TB type (non-MDR or MDR TB)
- *a_ik_* = fraction of infected people of type k within age group *i*
- *n_ijk_* = number of monthly contacts between individuals in age group *i* with TB type *k* and susceptible individuals in age group j = 30.5 days * *C_ij_*
- *C_ij_* = average number of daily physical respiratory contacts between age groups *i* and *j* defined in the contact matrix depicted above
- *p* = probability of transmission per contact, which is determined via calibration

This provides a model-generated rate of infection in age group *j* which is used to simulate transmission. Each healthy person in age group *j* is exposed to infection by non-MDR TB, then MDR TB. Because the probability of TB transmission given a physical contact (*p*) is not well established in the literature and is uncertain, we determined this value via model calibration (see Calibration section below).

*TB Activation*

Transmission results in latent TB infections. In the model, these infections may activate to become active TB due to “fast” or “slow” progression, where fast progression is defined as activation within 2 years of infection acquisition. Since the parameters associated with activation are uncertain, we calibrate the overall level of activation while using age specific relative risk of activation derived from from Horsburgh [11]. We use other sets of input parameters related to latent TB activation derived from the literature in the sensitivity analysis such as those from Vynnicky and Fine [12] (see Effect of Alternative Activation Parameters section).

*TB Treatment*

In addition to the natural history model of TB, the microsimulation also includes models of the national Indian TB treatment programs, DOTS and DOTS-plus, as well as private treatment. Below we describe the public sector treatment program ramp up, diagnosis, public sector treatment, and private sector treatment.

*TB Treatment: Public Sector TB Ramp up*

The model scales up DOTS and DOTS-plus programs according to historically observed patterns. This implies that in years between 1996 and 2006 certain portions of the population do not have access to DOTS. Between 1996 and 2006, we linearly interpolated coverage between years where coverage was reported (Figure S7 Panel A), until full population coverage was reached.


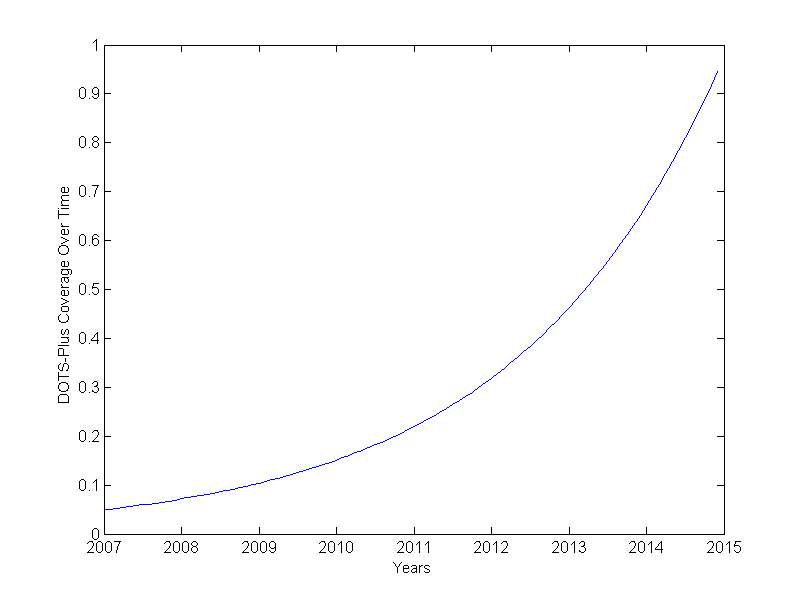

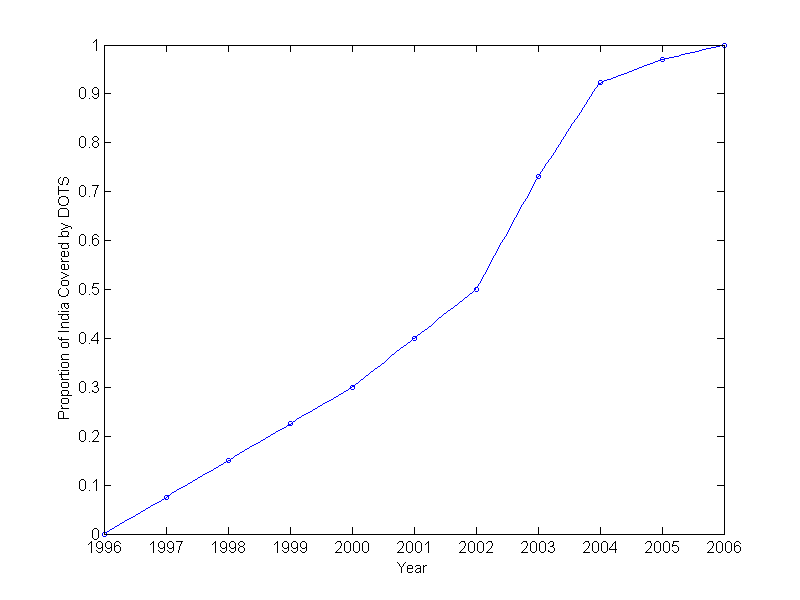


Figure S7: DOTS and DOTS-Plus Coverage Levels. Panel A: DOTS coverage used in the simulation. Full coverage used after 2006. Blue points represent reported coverage levels. Panel B: DOTS-Plus ramp up used in the simulation. An exponential function was fit to the data to provide monthly DOTS-Plus coverage until 2015, after which full coverage was assumed.

As DOTS-Plus was initiated in India in 2007 and has yet to reach full population coverage, we assumed that, consistent with current plans, full national coverage of DOTS-Plus will be achieved by 2015 [13]. As of 2011, only 26% of the population was reported to be covered by DOTS-Plus [13]. For future years through 2015, we assume DOTS-Plus coverage increases exponentially until full coverage is achieved in 2015, fitting an exponential function to observed coverage levels through 2011 (Figure S7 Panel B). Alternative DOTS-Plus ramp up schedules are explored in the Effect of Missing MDR Treatment Coverage Targets section.

*TB Treatment: Diagnosis*

Empirical studies in India have found that some individuals seek care only in the private sector while others first seek care in the private healthcare sector to varying degrees before ultimately being treated under public sector RNTCP TB treatment programs. We modeled this population heterogeneity in treatment seeking: a proportion of those individuals with active disease seek care only in the private sector while others may ultimately seek care through RNTCP. This behavior is consistent with published reports [14,15].

**
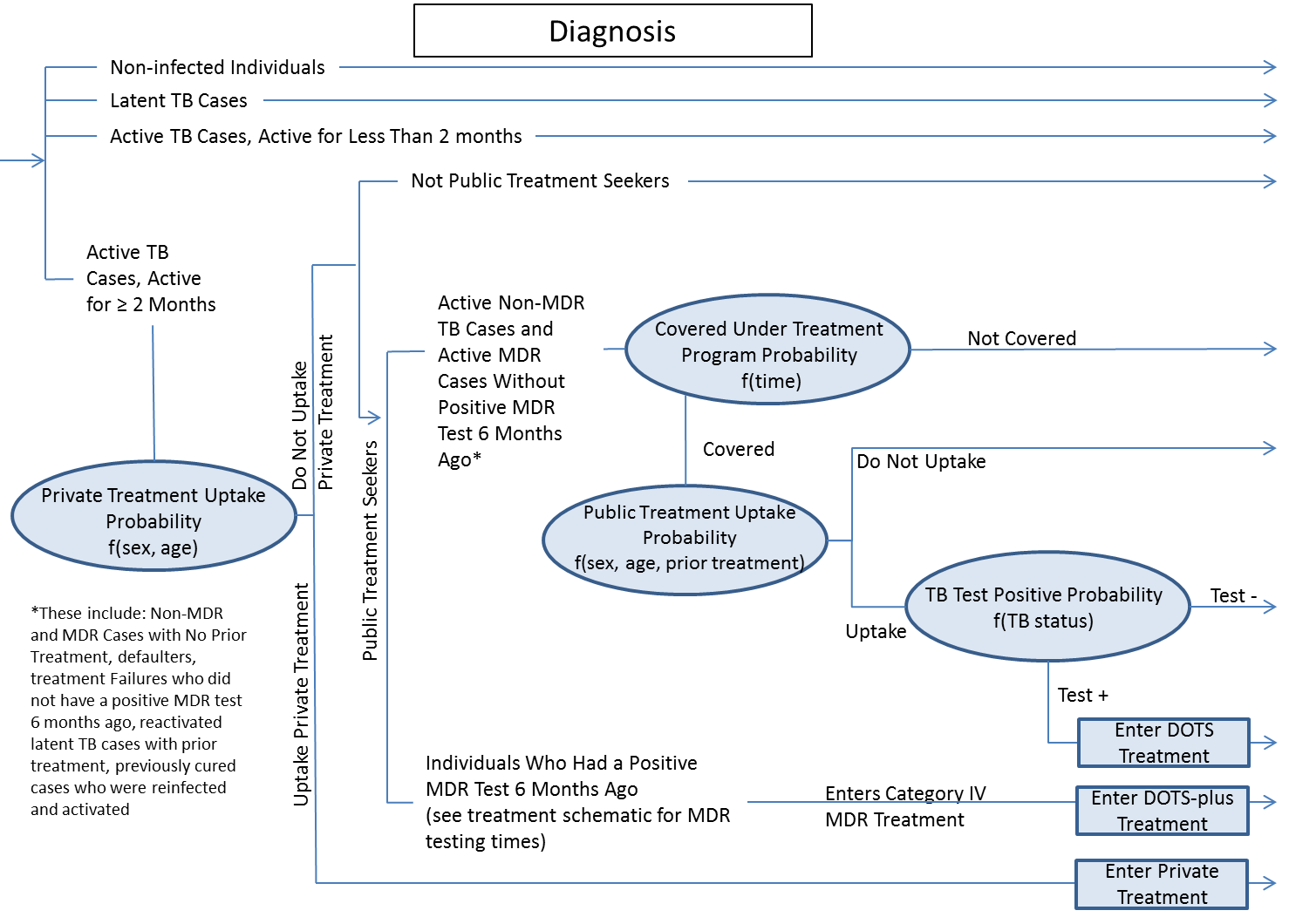
**

Although individuals may seek care, not all will receive care. Individuals with active disease (non-MDR or MDR) who seek care in the public sector can enter treatment if treatment is available and their triple sputum smear tests positive [16], with a probability determined by observed age-, sex-, and treatment category-specific trends calibrated to match overall treatment uptake levels in India in 2010. In order to simulate the minimum delay between activation, symptom onset, and treatment seeking, treatment is delayed until two months after activation for individuals who are probabilistically determined to enter treatment before that time. Overall delay from activation to treatment is on average about 6 months, which is consistent with the literature on delays to treatment after symptoms arise. [14,17–19] Individuals with prior DOTS treatment enter Category II treatment; all others entered Category I/III, unless drug sensitivity testing has confirmed MDR TB, in which case they enter Category IV treatment (Figure S8).

Figure S8: Detailed Model Schematic: Diagnosis

All individuals with active TB who have not been treated in the RNTCP system can seek private clinic care. Private clinic care-seeking behavior followed the sex- and age-specific uptake patterns of public sector treatment, but was scaled to be consistent with the number of private clinics RNTCP patients report visiting prior to entering the RNTCP system. [14]

*TB Treatment: Public Sector Treatment*

After entering treatment, every month individuals can die or default from treatment. Individuals who default discontinue treatment without cure and return to an active infectious TB health state. Patients with MDR TB in non-MDR TB treatment are subject to non-treatment mortality rates since treatment does not affect cure. At the end of the treatment regimen (6 months for Category I/III, 8 months for Category II, and 24 months for Category IV/DOTS-Plus), individuals are either successfully cured (no longer infected or infectious), have suppressed TB (latent TB, and thus no longer infectious but still infected with the potential to activate again in the future), or fail treatment with active TB (remaining both infected and infectious). Death, default, and treatment failure rates are age-, sex-, and treatment category-specific [2,20–23].

All treated patients with non-MDR TB who are not cured have a chance of developing MDR TB. We assume that treatment uptake does not select for or against patients with MDR TB as opposed to non-MDR TB. The mortality and treatment uptake in our model assumes this as well. Based on these assumptions, we used MDR TB information from Category II patients to estimate the MDR acquition rates among those defaulting or failing treatment. We use the Sharma study, which breaks down MDR-TB rates by patient type and also reports numbers consistent with nationally representative figures: the WHO reports the prevalence of MDR TB among previous treated TB cases to be 17.2% (95% CI 14.9%–19.5%) [24]. While this calculation does not adjust for individuals entering category I treatment already with MDR TB and hence may overestimate the rate of MDR TB acquisition from failed treatment, the results of our calibration that involve individuals entering treatment at various time points over more than a decade demonstrate reasonable consistency with the available empirical data on MDR TB in 2008 (see Face Validation section). This provides additional support that these estimates of MDR TB acquisition are reasonable.


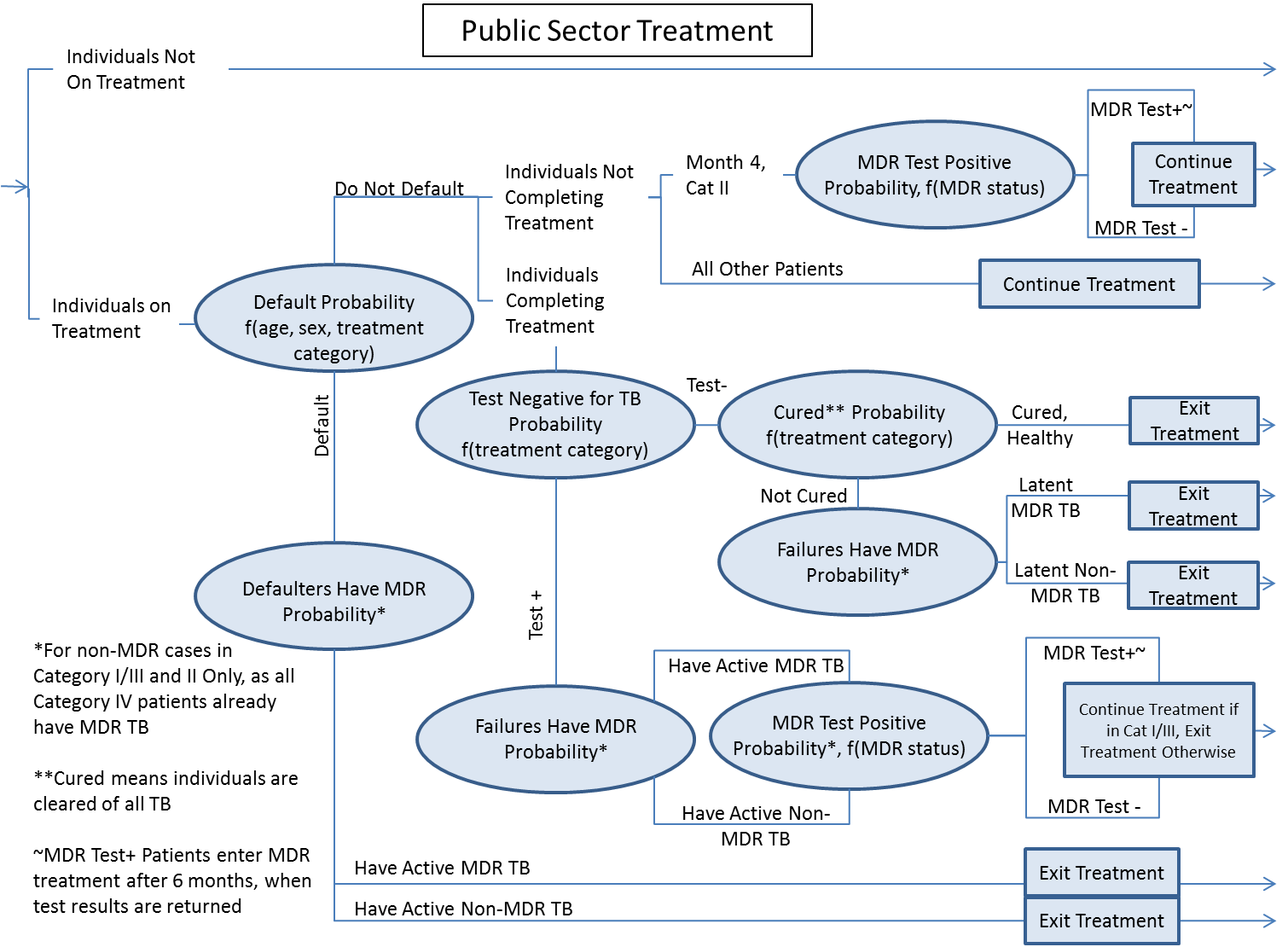


Figure S9: Detailed Model Schematic: Public Sector Treatment

Treatment outcome probabilities for each treatment category (i.e., I/III, II) were derived from RNTCP 2010Q3 reported national death, default, and failure rates [20]. To stratify these probabilities by age and sex, we used data from the RNTCP data and the literature and solved a system of equations [15,16]. The system of equations for category I/III used the odds ratio of male to female defaults, the sex and age breakdown of patients, and the overall default probabilities as reported in these sources. In order to simplify our calculations, we assumed that age-specific treatment default probabilities and sex-specific treatment default probabilities were independent.

The system of equations for stratifying default by sex is as follows (age stratification was performed similarly):

Odds ratio of male to female defaults = (A / B) / (C / D)

Proportion of males in treatment = (A + B) / (A + B + C + D)

Overall default probability = (A + C) / (A + B + C + D)

Total number of people = A + B + C + D

where:

A = number of males defaulting

B = number of males not defaulting

C = number of females defaulting

D = number of females not defaulting

We solved the system for A, B, C, and D; the solutions were then were used to compute the probabilities needed in the model. This procedure was repeated using category II data to find the corresponding category II treatment parameters, though due to a lack of data, we made the assumption that the odds ratios for defaulting (for both sex and age) were the same as for category I/III treatment. MDR TB treatment (DOTS-Plus or Category IV) is not stratified by age or sex due to extremely limited data on treatment outcomes for MDR TB in India.

Individuals who fail Category I/III treatment are put on Category II treatment. However, individuals who fail Category II treatment with active disease are not offered further treatment unless they test positive for MDR. At the end of all DOTS treatment categories and at the fourth month of Category II treatment, patients with positive sputum smears undergo drug sensitivity testing (MDR testing) for MDR TB using Lowenstein-Jensen culture on egg-based media, which is often considered a gold standard and therefore assumed to have 100% sensitivity and specificity for MDR TB.[25] If the sample taken at that time tests positive for MDR TB, they are ultimately placed into the MDR treatment program, Category IV (DOTS-Plus). Given typical times of sample transport, processing, results return, and notification, the delay between DST and initiating MDR treatment is 6 months, a conservative estimate given estimates of delay times.[26] Our model of public sector treatment is illustrated in Figure S9.

Individuals on appropriate treatment are not considered infectious; that is, non-MDR TB cases cannot transmit when on non-MDR treatment. However, MDR TB cases can still transmit when on non-MDR treatment, but not when they are on MDR treatment. Table S1, shows the number of individuals by age group, TB status, and public treatment status.

**Table S1: Number of Individuals in Model in 2011, by Health and Treatment Status**

|  | **Total Individuals** | **# with Active TB** | **# of Untreated Active TB** | **# in Cat. I Treatment** | **# in Cat. II Treatment** | **# in Cat. IV Treatment** | **% of Active TB Cases In Any Treatment** |
| --- | --- | --- | --- | --- | --- | --- | --- |
| Aged 20 and under | 573,383,065 | 951,618 | 783,755 | 104,134 | 41,297 | 22,431 | 17.64% |
| Aged 21-60 | 569,847,104 | 2,763,495 | 2,242,378 | 356,077 | 112,453 | 52,587 | 18.86% |
| Aged 61 and up | 81,383,831 | 403,910 | 331,120 | 49,765 | 15,598 | 7,428 | 18.02% |
| **Total** | **1,224,614,000** | **4,119,023** | **3,357,253** | **509,976** | **169,348** | **82,446** | **18.49%** |

*TB Treatment: Private Sector Treatment*

After entering private clinic care, individuals are exposed to a one-period risk of developing MDR TB from inadequate treatment, as the duration individuals spend with each private clinic is between 2-4 weeks. The probability of developing MDR in a private clinic was assumed to be identical to that of a defaulter in the RNTCP system. TB cure rates in private clinics not using DOTS regiments are likely to be very low [27–29]; for simplicity, we assume that TB cure rates in private clinics are zero. Individuals have the possibility of repeatedly seeking private clinic care up to a maximum of seven private clinic treatment exposures. This tracks the distribution of private providers seen by TB patients in India as documented in the literature. Our model of private sector treatment is illustrated in Figure S10.


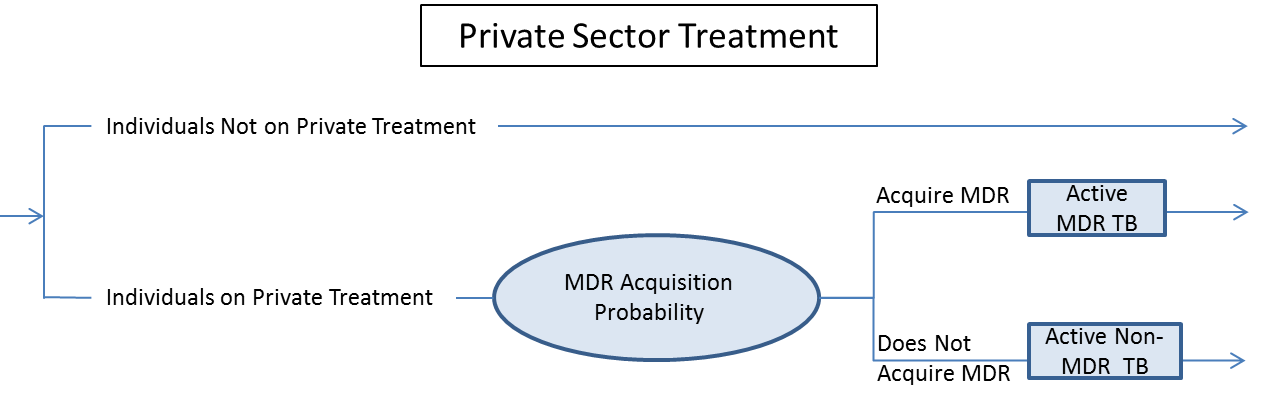


Figure S10: Detailed Model Schematic: Private Sector Treatment

*Calibration*

We used model calibration to infer several input parameters required by the model. These are parameters that are particularly uncertain and for which no studies have directly measured in India. Model calibration involves systematically varying unknown or uncertain model inputs until model outputs match empirically observed data. In our case, we performed model calibration for these parameters using a hierarchical random grid search over plausible value ranges for each parameter to produce output values simultaneously consistent with many measures of observed data in terms of their point estimates and corresponding confidence intervals.

We first calibrated two parameters related to overall TB: 1) an activation rate that determines the average time to activation for individuals with latent TB infections; and 2) the effective contact rate, a parameter that determines the average probability of TB transmission given a contact between a susceptible and infectious individual. We used calibration to determine the underlying level of activation and transmission while preserving age-specific relative probabilities provided in the literature [6] [11]. The activation rate parameter was varied from 0 to 4, with a step size of 0.03, and the transmission rate parameter varied from 0 to 0.0030 with step size of 0.0001. Not all points in the space were sampled; higher activation scaling factors would generate higher incidence and prevalence trends, holding the transmission parameter constant, and vice versa, so combinations of parameters that would not move the incidence and prevalence in the correct direction were not examined. These ranges were chosen because parameters outside these intervals generated outputs far outside the target range. We used active and latent TB estimates from the literature [3] in 1999 (see Table S2), and incidence and prevalence estimates from 1996-2000 from the WHO as our output targets -- these are shown in figure 2 of the main manuscript. We chose final parameters (activation parameter of 2.16 and transmission parameter of 0.0022) such that the maximum number of simulation output points lay within the WHO uncertainty bounds (for both incidence and prevalence), with preference towards parameter sets that generated outputs closer to the mean if multiple parameter sets generated outputs with the same number of points within the uncertainty bounds.

We then calibrated DOTS treatment uptake probabilities -- the probability of an individual with active TB living in an area with RNTCP treatment to go to a clinic, get tested for TB, test positive for TB, and enter treatment. These are stratified by age and sex, and we used the same search method described above to generate model outputs consistent with RNTCP 2010Q3 overall treatment usage and patient demographic data [20]. The development of active TB disease was simulated in the model with levels of treatment coverage, test sensitivity, and test specificity derived from the literature [16] [30]. However, an individual’s probability of undergoing TB testing given his or her development of active disease is not reported in the literature and can depend on poorly measured factors such as knowledge of TB symptoms, knowledge of TB treatment, ease of accessing treatment, and social stigma of acknowledging having active TB. From reported treatment demographics, we know TB treatment uptake varies by age and sex [22]. To find treatment uptake levels, we used the age- and sex-specific proportion of patients enrolled in the first month of treatment from the literature [21]. Then we calibrated the average probability of undergoing TB testing among individuals with active disease while holding the previously-calibrated transmission and activation rates fixed. Similarly to the process above, we varied DOTS treatment uptake probabilities by changing the probability of being tested for TB for individuals with and without prior treatment (between 0 and 0.002000, with step size of 0.000025 for both with and without prior treatment). As before, not all points in the space were sampled because a higher probability of being tested for TB generated a higher number of patients registered in DOTS treatment, so parameters that would move the simulation outputs in the wrong direction relative to the targets were not sampled. The output targets for treatment are given in Table S2. Parameters that generated simulation outputs closer to the targets were preferred. Final values of 0.000850 and 0.001275 were used for the base case, for treatment-naïve and not-treatment-naïve, respectively.

**Table S2: Treatment Uptake Targets**

|  | **Empirical Estimate** | **Simulation Estimate** | | **Source** | |
| --- | --- | --- | --- | --- | --- |
| **Prevalence and Incidence** |  |  |  | |  |
| Proportion of population with latent TB, 1999 | 0.44 | 0.5005 | [3] | |  |
| Proportion of population with active TB, 1999 | 0.005 | 0.006 | [3] | |  |
|  |  |  |  | |  |
| **Treatment Uptake** |  |  |  | |  |
| Proportion of total population registered 2010Q3 | 0.000313 | 0.000290 | [20] | |  |
| with no prior treatment | 0.000263 | 0.000224 | [20] | |  |
| with prior treatment | 0.000064 | 0.000066 | [20] | |  |
| Ratio of new registrations of new- to prior-treatment status individuals | 4.07 | 3.410488 | [20] | |  |

After calibration, model outputs were simultaneously consistent with a variety of demographic and epidemiological data in the literature (see subsection Internal and Face Validation). Notably, TB prevalence and incidence were within the range of WHO reported time trends [4].

In addition to calibrating to pre-DOTS latent and active TB in India and to treatment uptake demographics listed in the table above, the model was also required to be simultaneously consistent with time-trends in active TB prevalence and incidence between 1996 and 2012 and to MDR TB-specific values reported for 2008 [24]. These latter calibration results are shown in Figure 2 of the main paper.

*Analysis Scenarios*

In this paper, we are concerned with the implications of India’s transitioning MDR epidemic: an increasing proportion of new MDR cases are transmission-generated, as opposed to treatment-generated. We explore the impact of this transition on the effectiveness of two types of control strategies: one that primarily reduces MDR through improving treatment quality to limit strain selection toward MDR, and one that primarily reduces MDR through promptly identifying MDR cases and providing effective treatment to limit transmission. We examined the effects of starting each policy in 1997, 2007, 2017, and 2027 in order to establish the time-varying effect of each policy as the MDR epidemic changed. We used the ten-year average percentage reduction in MDR prevalence as our outcome measure, so scenarios were spaced ten years apart. The 2017 and 2027 scenarios have particular bearing on future policy as they show the impact of delaying the different types of strategies.


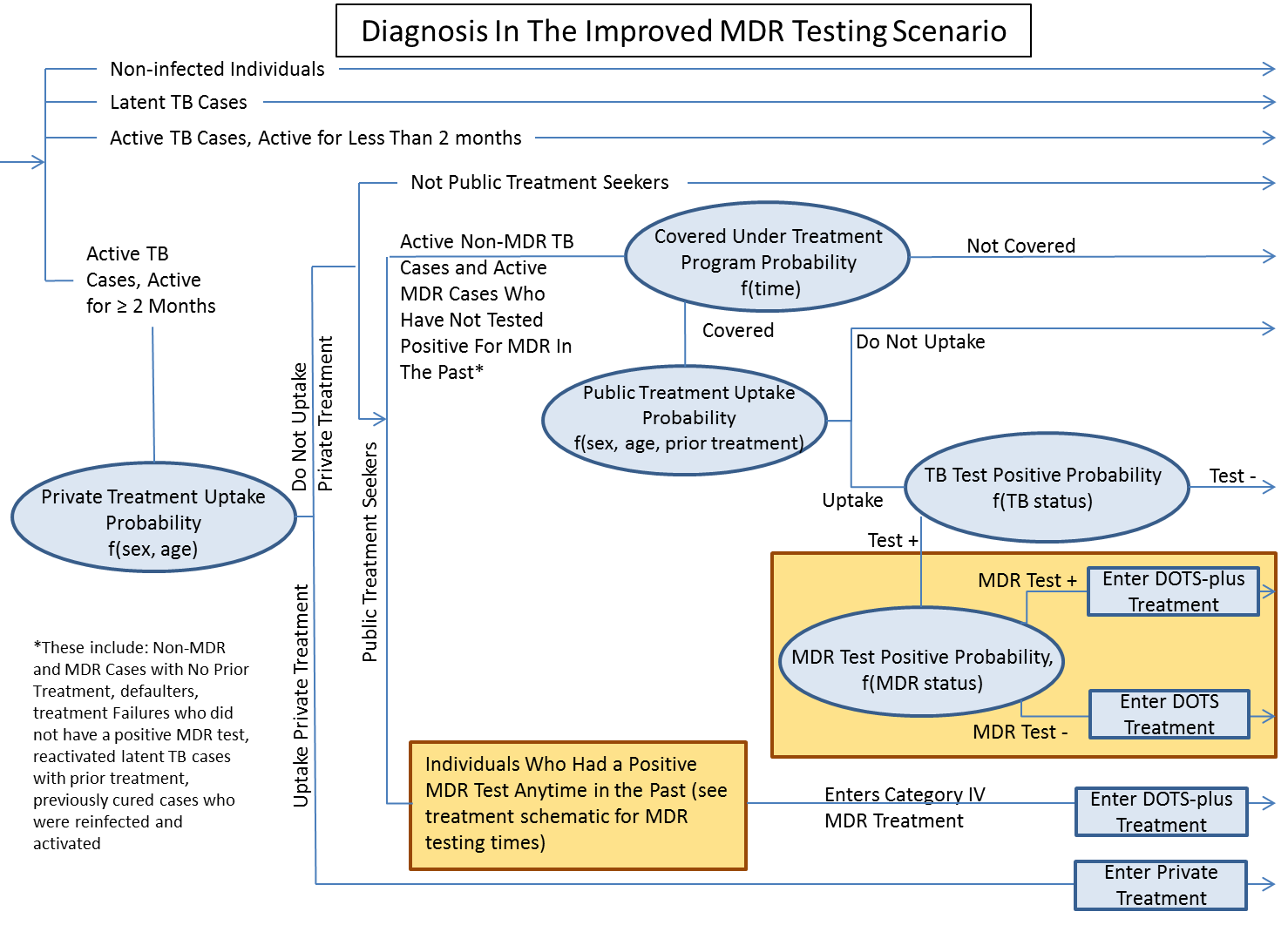


Figure S12: Diagnosis in the Improved MDR Testing Scenario. Differences from the base case are highlighted in yellow. An additional MDR testing is done in the first month of treatment and those found positive for MDR are sent immediately to MDR treatment

In our examination of treatment quality improvement to limit strain selection towards MDR, we assumed improved DOTS treatment in which default, failure and death rates were lowered to the best observed across Indian states in 2010. These parameters, shown on Table S5.8, were calculated using the same method as described above in the Treatment section.

For our scenario to reduce MDR through promptly identifying MDR cases and providing effective treatment to limit transmission (Figure S12 and S13), we modified the existing diagnostic and treatment algorithms to include rapid drug sensitivity testing. Diagnosis continued to use the three-sputum smear tests but in the modified algorithm also administered drug sensitivity testing in the first month of treatment to all patients (instead of at the end or mid-way through treatment). MDR testing specificity and sensitivity remained the same as in the base case. A patient who tested positive for MDR was immediately placed on appropriate MDR treatment if he or she was covered by DOTS-Plus. This increased the number of patients appropriately in MDR treatment (since patients had a much smaller probability of default prior to MDR testing) and also reduced the time MDR patients were infectious since MDR treatment prevents MDR transmission.

We also examined a combined scenario where both treatment quality and MDR testing were improved together. This allowed us to characterize the synergistic effects of the policies.


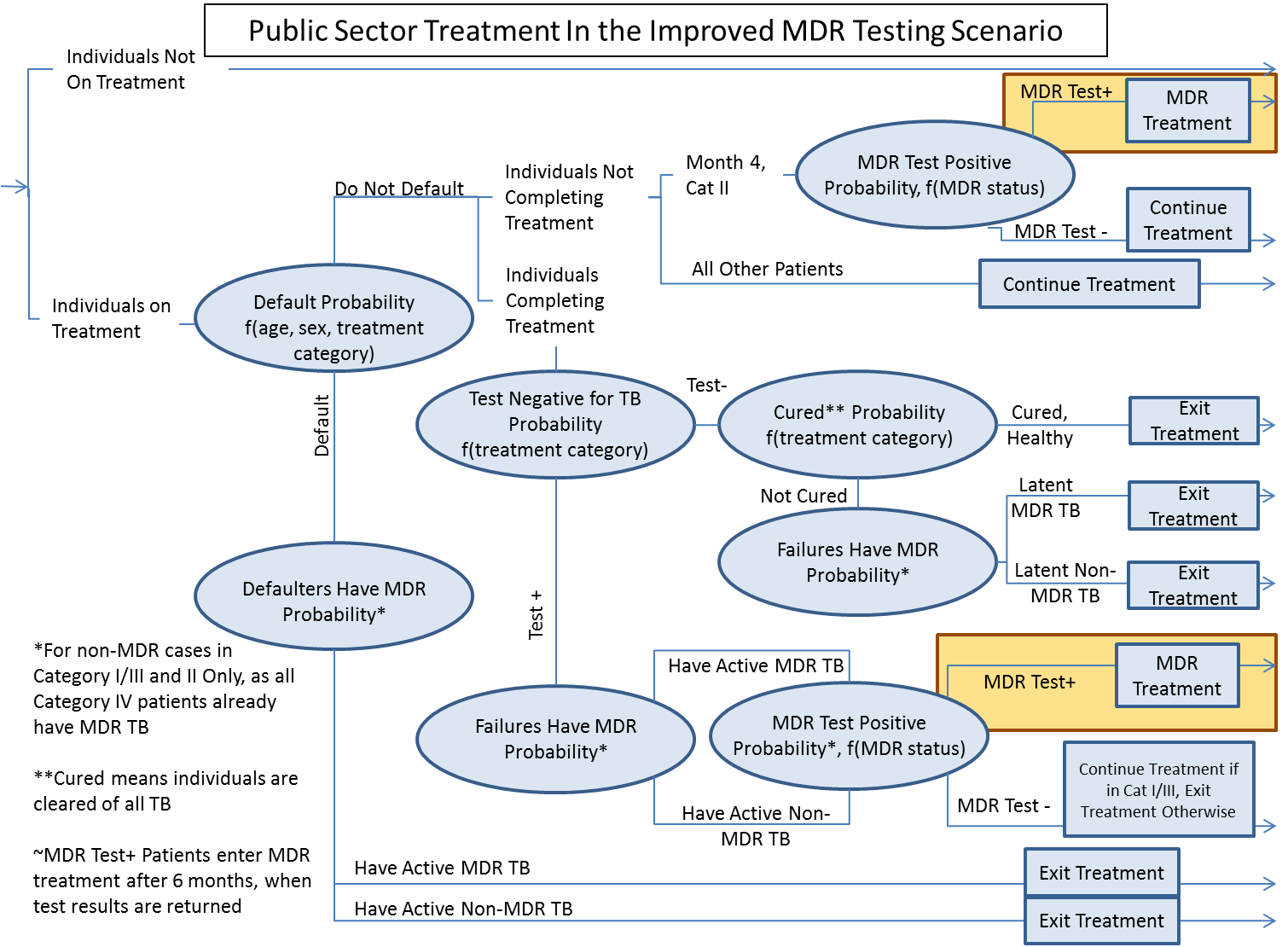


Figure S13: Treatment in the Improved MDR Testing Scenario. Changes are highlighted in yellow. Those who test positive for MDR are sent to MDR treatment in the next month, instead of waiting for 6 months as in the base case.

*Treatment-Generated and Transmission-Generated MDR TB*

We differentiate between treatment-generated and transmission-generated MDR cases. Treatment-generated cases are defined as those cases of active MDR TB disease that arise from treatment default or unsuccessful treatment. If an individual who was infected with non-MDR TB fails treatment and the infection becomes MDR, the case is counted towards treatment-generated MDR incidence rates at the time of activation. A transmission-generated case of MDR TB is due to contact with and transmission from an infectious MDR TB case and the newly infected individual is counted towards transmission-generated incidence if and when that person’s infection activates.

Our definitions of transmission-generated and treatment-generated cases are distinct from WHO definitions of “incident new and relapse” and “incident acquired MDR TB cases.” Our model provides a direct measurement of MDR cases arising due to treatment and due to contact with an individual with active TB; the WHO’s measures are estimates derived from available observed data.

**Appendix Section 2: Internal and Face Validation**


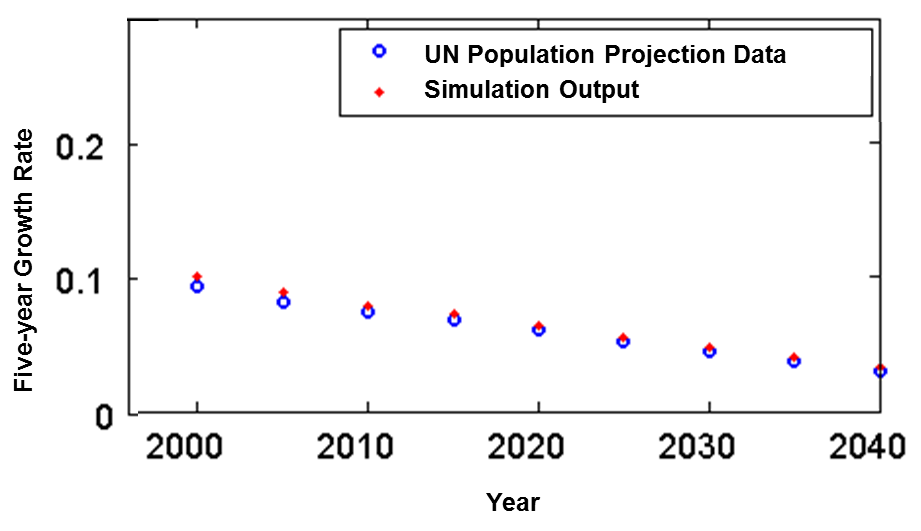
After calibration, we confirmed that the model continued to perform as expected in our internal validation on measures such as population growth and treatment quality measures. Population growth in the model was consistent with UN trends [1], shown in red in Figure S11. Model inputs used UN population data, but population growth was also influenced by modeled mortality.

A number of additional model outputs were consistent with the observed data as shown in Table S3. Life expectancies of both males and females simulated under 1990 conditions had confidence intervals close to or overlapping WHO reported values. In an external validation, model outputs on the annual risk of TB infection (ARTI) in India for children age 1-9 were compared with those reported in Chadha et al [31]. Reproducing the ARTI estimation procedure described in the article, the model predicted a 5.3% decline in the national ARTI/year over the study period (2000-2010), consistent with the 4.5% (95% CI 2.8% to 6.3%) calculated in the Chadha et al.

Figure S11: Comparison of Model (red) to Observed and Predicted Growth Rates (blue) as Reported by the United Nations.

There is some uncertainty in the literature around the life expectancy after TB activation and the fraction of latent cases that activate over a lifetime. Model outputs were close to reported estimates. Other measures of TB epidemiology in the model, such as latent and active TB prevalence, were also similar to reported values. A considerable amount of uncertainty surrounds the number of secondary TB infections per smear-positive person-year, which can differ by population density, cultural practices, and other factors; nonetheless, the model produces a value close to those from other TB models in the literature [32]. For individuals with treated TB, treatment death and default rates are consistent with those reported by the RNTCP in 2010 [20] for both category I/III and II.

**Table S3: Additional Internal and Face Validation Results**

|  | **Empirical Estimate (95% CI, if available)** | **Simulation Estimate (95% CI)** | **Source** |
| --- | --- | --- | --- |
| **Demographics** |  |  |  |
| Male life expectancy, 1990 (years) | 57.2 | 55.9 (55.6 - 56.2) | [2] |
| Female life expectancy, 1990 (years) | 57.9 | 55.7 (54.3 - 57.1) | [2] |
|  |  |  |  |
| **Tuberculosis** |  |  |  |
| Life expectancy post TB activation   without treatment (years): | ~3 years | 3.11 (2.95 - 3.28) | [33] |
| Lifetime fraction of latent infections that  activate: | 0.10 - 0.20 | 0.17 (0.15 - 0.18) | [11] |
|  |  |  |  |
| **Treatment** |  |  |  |
| Average delay from symptom onset to RNTCP treatment (months) | 2 to 7 | 6.1 (5.63-6.57) | [14,17–19,34] |
| Median delay from symptom onset to RNTCP treatment (months) | 2 to 7 | 3.9 (3.72 – 4.08) |  |
|  |  |  |  |
|  |  |  |  |
| Default rates: |  |  |  |
| Category I | 0.06 | 0.054 (0.047 – 0.061) | [20] |
| Category II | 0.14 | 0.151 (0.119 – 0.183) | [20] |
|  |  |  |  |
| **MDR Incidence in 2008 Using WHO Calculation Methods** |  |  |  |
| % MDR among new TB cases | 2.3 (1.8-2.8) | 2.3 (2.1 - 2.5) | [24] |
| Number of MDR-TB among incident new and relapse TB cases | 55,000 (40,000-74,000) | 52,000 (47,000-56,000) |  |
| Number of incident acquired MDR-TB cases | 43,000 (33,000-56,000) | 73,000 (52,000-94,000) |  |
| Number of MDR-TB among incident total TB cases | 99,000 (79000-120000) | 124,000 (99,000-150,000) |  |


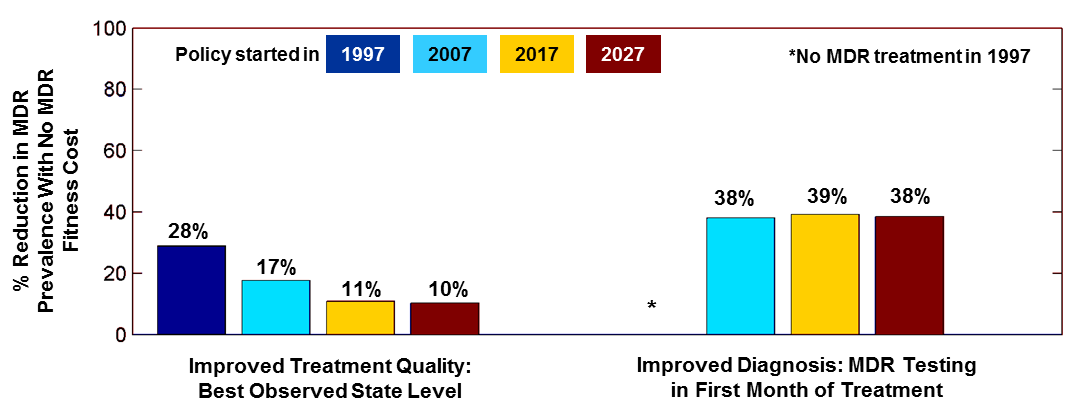
**Appendix Section 3: Sensitivity Analysis**

In the following section we present the effects of potential model changes and scenarios. We replicate the main results in Figure S14 for ease of comparison. In the base case, DOTS remains at full scale up and treatment quality, DOTS-Plus scales up as planned to nationwide coverage, and private clinics do not cure non-MDR TB and may cause MDR TB. As in the main paper, the outcome measure of interest is the percent reduction of infectious MDR TB (the prevalence of MDR TB cases not on effective treatment) over ten years. We explore two policies: improving the non-MDR TB treatment quality and improving the rapidity of MDR TB diagnosis. We present the outcome of both policies if they were implemented starting in 1997, 2007, 2017, and 2027 to illustrate how delays in implementation may change policy effectiveness.

Figure S14: Base Case Results

*Effect of Missing MDR Treatment Coverage Targets*

Given that current population coverage of MDR treatment is well below full nationwide coverage, and that expansions planned through 2015, we considered the effectiveness of the MDR control scenarios described above if the planned expansions did not occur. MDR treatment is a key component in the reduction of MDR TB. Unlike non-MDR treatment that has already achieved nationwide coverage, levels of MDR treatment coverage were 26% as of 2011 [13]. Holding MDR treatment coverage at this level through 2038, we repeated our scenario analyses and computed the average percentage reduction in infectious MDR TB prevalence in the ten years after policy initiation, using a comparator where the improved treatment quality and improved MDR testing policy were never begun. Results are shown in Figure S15.

Figure S15: MDR Treatment and Scenario Effectiveness


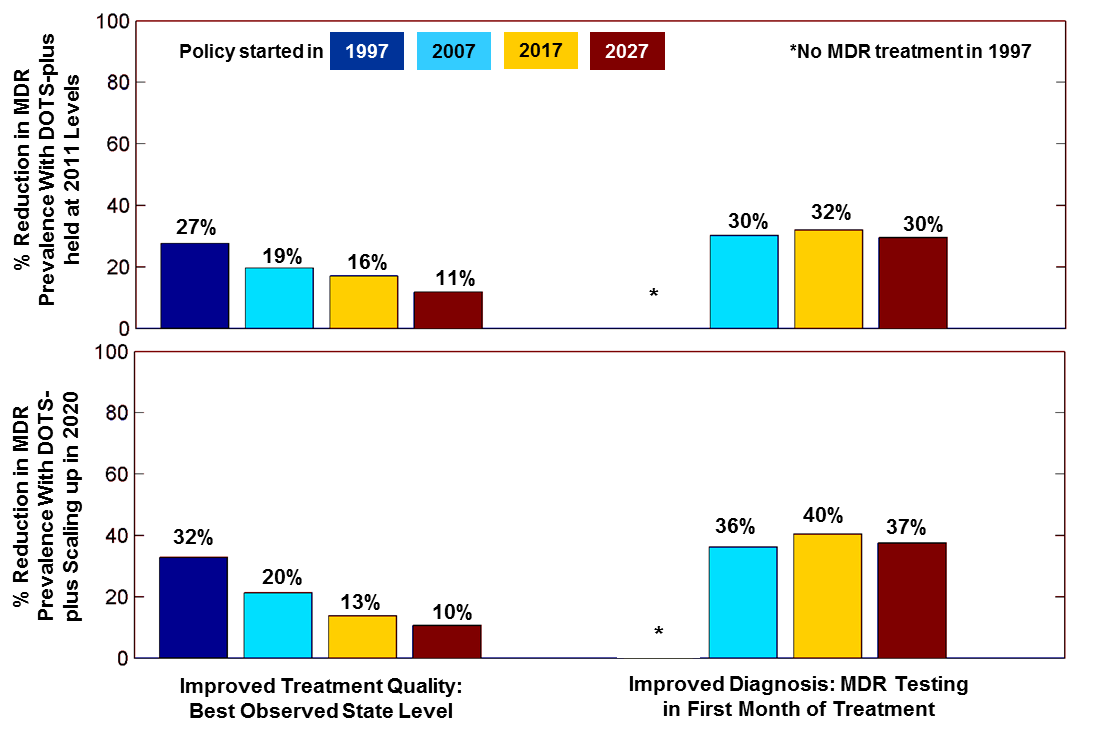
The general pattern of constant effectiveness of MDR diagnosis and declining effectiveness of improved non-MDR treatment remain consistent with the overall effect of the MDR epidemic transition (compare yellow bars and red bars within each panel of Figure S14). However, without full coverage, the reduction in infectious MDR TB prevalence resulting from improved MDR diagnosis is smaller. This is because even if MDR cases are identified, only those covered by an MDR treatment program can be effectively treated. The effectiveness of improved MDR testing is blunted when additional identified cases are not treated effectively. This also means that the benefit of preventing a treatment-generated case through improved non-MDR treatment is magnified, as each MDR case contributes more to the higher overall MDR prevalence through downstream chains of transmission. Therefore each MDR case averted by improvements to treatment quality represents a larger reduction in MDR prevalence, leading to the increase in percentage reductions in the improved treatment quality scenario (compare top left panel of Figure S15 to left panel of base case).

We also examined the effect of DOTS-Plus achieving full ramp up in 2020 (instead of by 2015, as scheduled). MDR treatment was assumed to ramp up linearly at 2011 levels starting in 2011. This did not change the proportion of new MDR TB cases that were transmission-generated, which increased as in the base case. The efficacy of improving treatment and improving MDR diagnosis showed the same patterns as before (bottom panel of Figure S15), with levels between MDR treatment held at 2011 levels and base case MDR treatment scale up scenarios, as expected.

*Effect of Differential MDR Transmission Fitness*


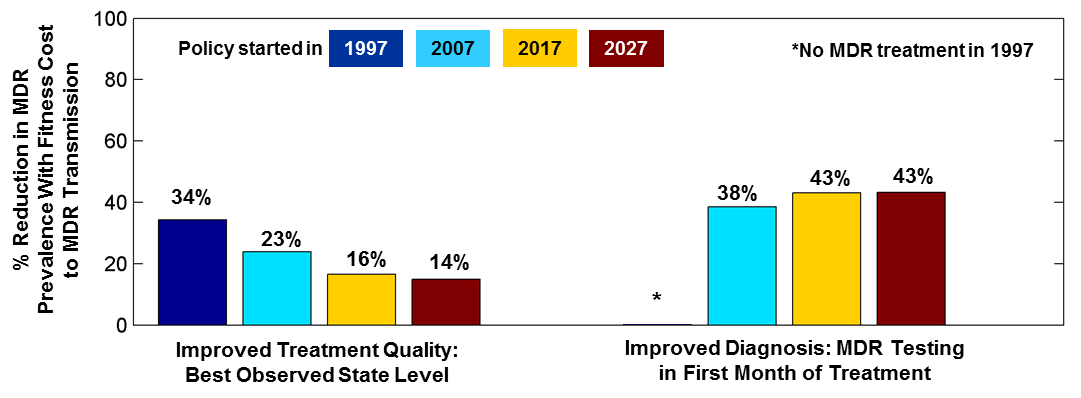
Some studies suggest that drug-resistant strains of TB may be less fit than non-drug-resistant strains [35–37]. We explored the impact of reduced MDR transmission fitness by reducing the probability of MDR transmission to 70% that of a drug sensitive strain. The percent reduction in MDR prevalence through treatment and diagnosis improvements is similar to that seen in the base analysis (Figure S16). As expected, overall levels of policy effectiveness are higher. Delays in starting policy improvements result in similar trends as in the base case, with smaller effectiveness associated with longer delays in improving treatment quality, and delaying policies that improve MDR diagnosis result in generally constant effectiveness. Because MDR cases are less transmissible, the total prevalence of MDR TB is lower and each averted case represents a larger percentage reduction in MDR TB prevalence. This results in larger overall effectiveness in both types of policies compared to the base case (Figure S14).

Figure S16: MDR Fitness and Scenario Effectiveness

**
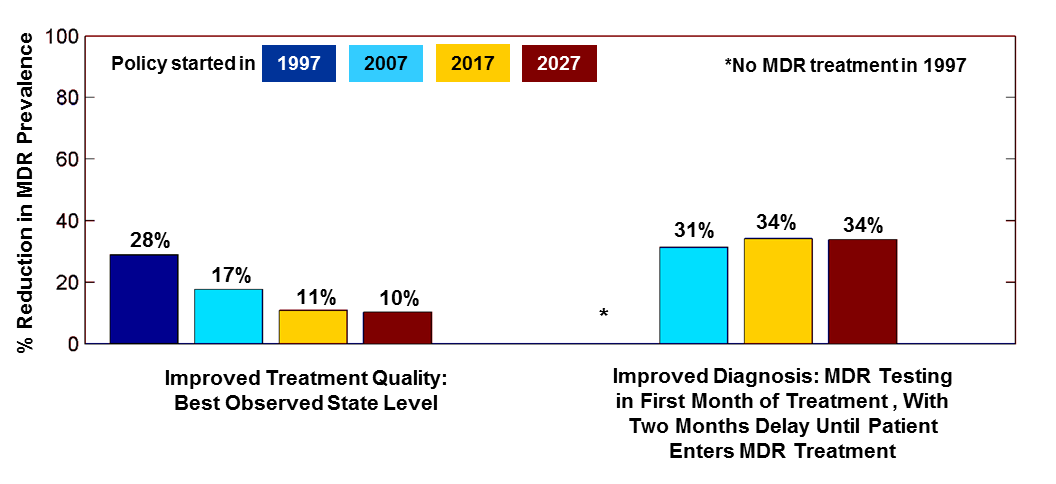
***Effect of Delay in MDR TB Treatment Even After Rapid MDR TB Diagnosis*

While rapid diagnostic test for MDR TB can provide results quickly, it is not certain that MDR treatment will be initiated immediately after diagnosis. This is for a variety of reasons including patients needing time to make final decisions, administrative delays, and differential availability of MDR drugs and personnel to initiate their administration. We considered how MDR treatment delays like these might attenuate the benefits from rapid MDR diagnosis in a sensitivity analysis. Specifically, instead of initiating MDR treatment within a month of MDR diagnosis (base case), we examined a scenario in which MDR treatment was initiated 2 months post MDR diagnosis and also compared this to current MDR diagnostic practice in which MDR treatment is initiated 6 months after MDR diagnosis. Results are presented in Figure S17. As expected, the percentage reduction in MDR prevalence is lower than in the base case (compare to Figure S14, right panel), but the time trend remains robust and rapid MDR diagnosis even with somewhat delayed MDR treatment still is more effective in reducing MDR prevalence than improving non-MDR treatment quality.

Figure S17: Delays in entering MDR Treatment and Scenario Effectiveness

*Effect of Private Clinics Achieving Cure for a Proportion of Non-MDR TB Patients*

The quality of care in private TB clinics in India is not well characterized and likely heterogeneous. While studies show that many TB patients seek care from unqualified providers who deliver non-standard TB treatment regimens prior to entering public sector TB treatment under the RNTCP, others may receive care from providers using effective regimens, never seek care in RNTCP, and therefore are never documented. We considered how our results may change if private clinics are able to achieve cure for a proportion of individuals with non-MDR TB that they **
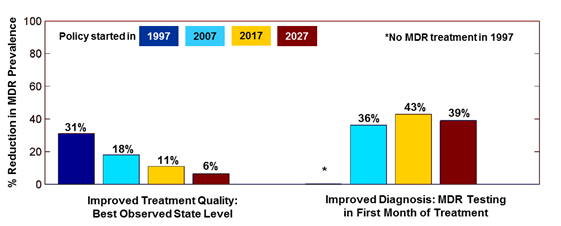
**treat in sensitivity analysis. Specifically, we examined a scenario in which private clinics were able to cure non-MDR TB patients at a monthly rate of 3%; this is equivalent to 22% of patients if they adhered to a full eight month regimen (the DOTS recommended duration) of the appropriate drugs compared to 0% in the base case (Figure S14). Demographic and disease outputs remained consistent with calibration targets and validation measures under this scenario. Results were similar to those in the base case, and the effects of the policy scenarios are presented in Figure S18.

Figure S18: Effect of Private Clinics Achieving Cure for Some Non-MDR TB Patients and Scenario Effectiveness

Figure S18: Private Clinic Cure Rates and Scenario Effectiveness

*Effect of Variation in Private Clinic MDR TB Generation Rate*

**
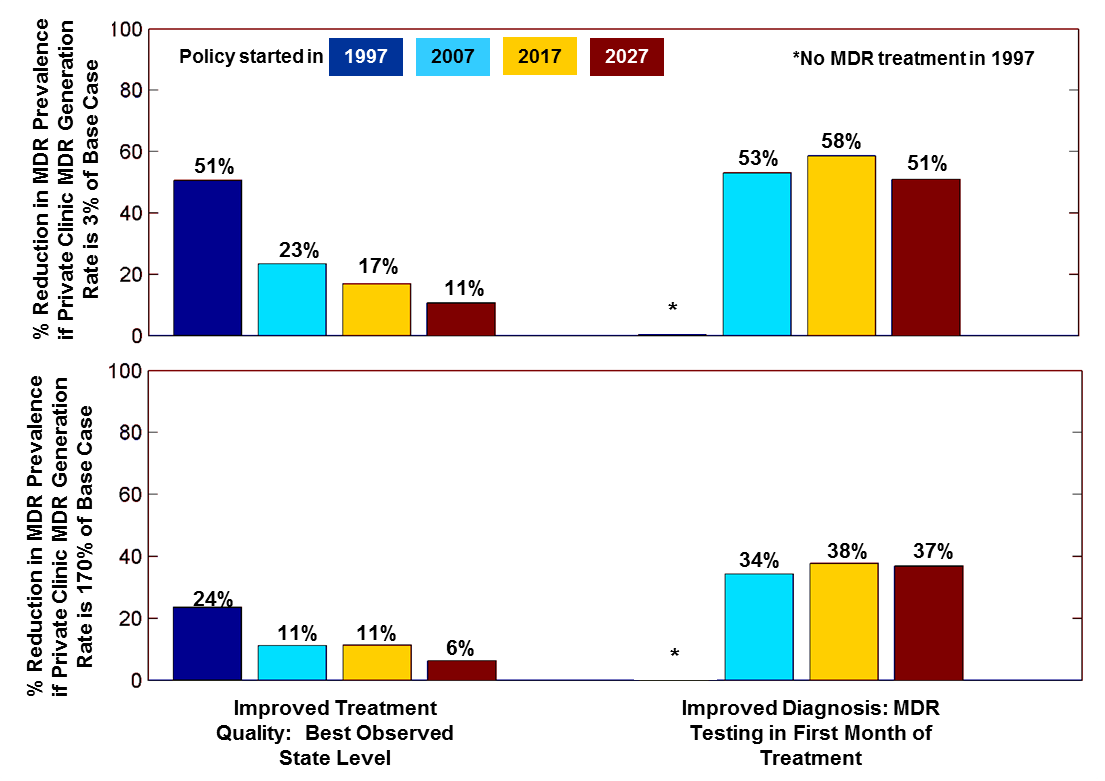
**Patients undergoing treatment in private clinics may be exposed to first line TB drugs but receive inadequate doses or durations of treatment, which could generate MDR TB. Since private clinic drug regimens and patient flows are poorly documented, the data surrounding private clinic MDR TB generation is scarce and uncertain. In our base case, we assume that private clinics generate MDR TB cases at the same rate as public clinics in Category II treatment. We explore the impact of changing that assumption on our policy effectiveness by varying the probability of monthly MDR TB acquisition in private clinics to be 3% or 170% of the base case.

Figure S19: Variation in Private Clinics MDR T Generation and Scenario Effectiveness

Figure S19: Private Clinic MDR TB Generation and Scenario Effectiveness

The model did not need to be recalibrated with this range of variation, as disease and demographic calibration targets and validation measures remained similar to those in the base case. The effectiveness of the treatment quality improvement and diagnosis improvement policies are presented in Figure S19. The time trends in both policies remain the same as in the base case (Figure S14). Policies that improve treatment quality decline in efficacy over time, while those that improve the speed of MDR TB diagnosis do not. As expected, the efficacies of both policies are higher if private clinics generated less MDR TB -- MDR TB control policies are more effective in an environment with lower MDR TB generation, as each averted MDR TB case is a larger percentage of the total MDR TB population.

*Infectious MDR TB Prevalence over Time*

A summary of infectious MDR TB prevalence under the scenarios described are summarized in Figure S20, with the base case in blue-green. Infectious MDR TB prevalence declined in 2006 when the DOTS-Plus MDR TB treatment program began (see decrease around that period in Figure S20) in all scenarios. At their most extreme, these scenarios generate a difference in MDR TB prevalence of 31 cases per 100,000 people, but all scenarios show the prevalence of MDR TB increasing over time.


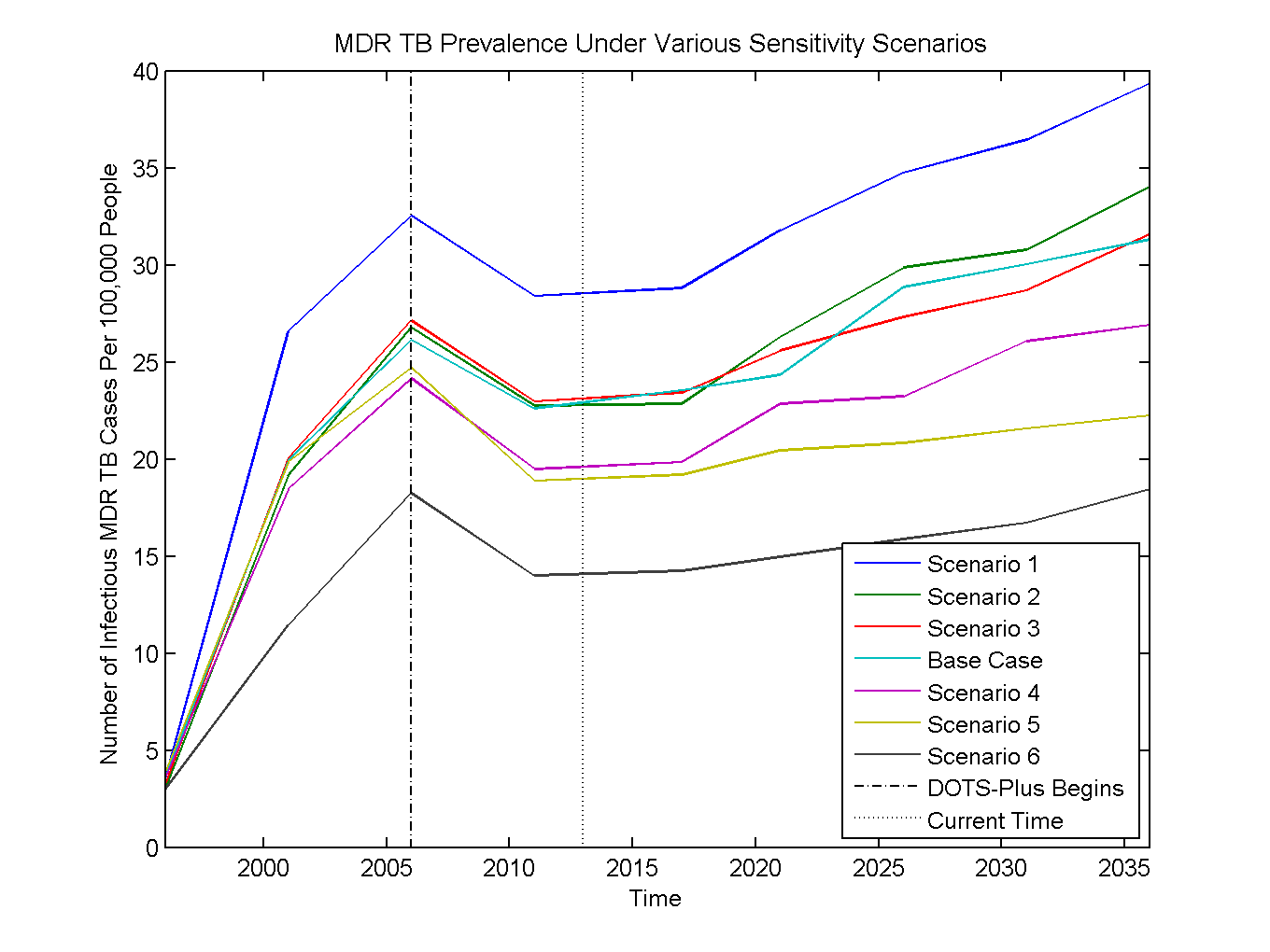


Figure S20: Number of MDR TB Cases Over Time In Various Sensitivity Scenarios In the Absence of New TB Interventions. Scenario 1 increases the private clinic rate of MDR TB generation to 1.7 times that of the base case. Scenario 2 holds DOTS-Plus levels constant at 2011 levels. Scenario 3 has DOTS-Plus achieving nationwide coverage in 2020. In scenario 4 private clinics cure a proportion of their non-MDR TB patients. Scenario 5 has MDR TB with lower transmission fitness. Scenario 6 private clinics generate MDR TB at only 3% of their rate in the base case.

This graph shows that some model assumptions may be more influential than others in terms of MDR TB prevalence. Increasing the MDR TB generation in private clinics to 1.7 times that of the base case generates an increase of approximately 8 cases per 100,000 from the base case, whereas scenarios that have DOTS-Plus missing scale-up targets have similar prevalence trends as the base case. When private clinics cure non-MDR TB, cured patients are not exposed to the risk of acquiring MDR TB in either private or public clinics, and this is reflected by a lower prevalence in MDR TB in this scenario. Unsurprisingly, this is also the case when MDR TB is less infectious due to lower transmission fitness and if private clinics generate 0.03 times the MDR TB generated in the base case.

Figure S21 gives the number of infectious MDR TB cases if policies to improve non-MDR treatment quality began in 2017. All scenarios show a decline in MDR prevalence of about 5 cases per 100,000 in 2038. The ordering and distance from the base case of the various scenarios remain consistent with those in Figure S20, without the policy.

Figure S21: The Effect of Improving Non-MDR Treatment Policies in 2017 Under Various Scenarios. The scenarios are described in the Figure S20 description.


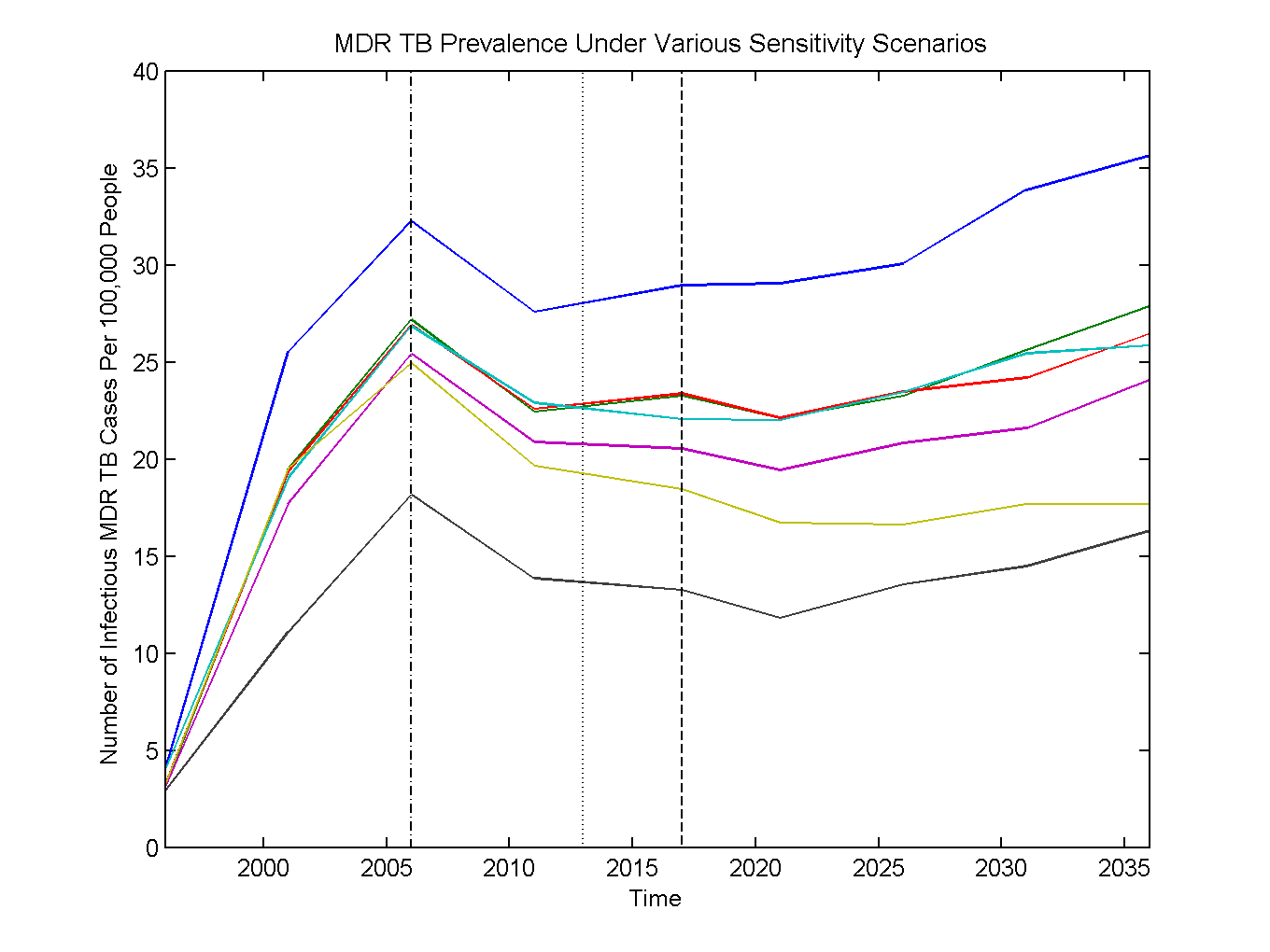

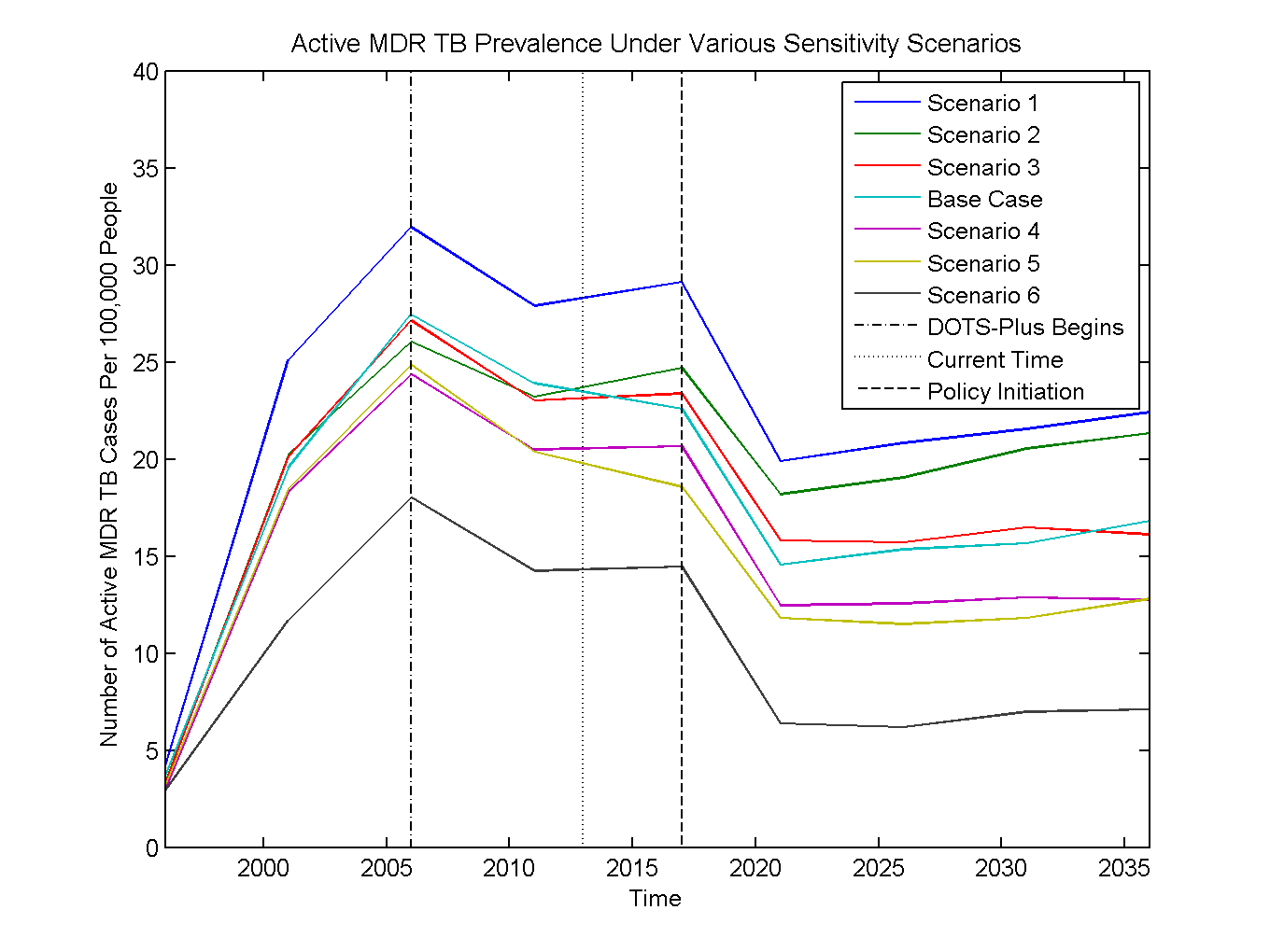


Figure S22 gives the number of infectious MDR TB cases if policies to improve MDR TB diagnosis began in 2017. MDR TB prevalence declines sharply after policy initiation but then levels out, resulting in a prevalence reduction of 11-22 cases per 100,000, depending on the scenario, compared to the base case (Figure S20) by 2038. Again, the relative ordering of the scenarios by prevalence is consistent with Figure S20, where no policy was implemented. However, the difference in prevalence levels decrease to about 15 cases per 100,000 between the most extreme scenarios (this was roughly 25 cases per 100,000 in Figure S20 and S21), indicating uncertainties in the model may not less influential when evaluating this policy’s outcomes.

Improving MDR TB diagnosis (Figure S22) may have different effects on absolute infectious MDR TB prevalence given assumptions about treatment and disease characteristics. Comparing the MDR TB prevalence trends with the policy to those without the policy, we see a larger prevalence gap between the base case and scenario 2, where DOTS-Plus coverage remains at 2011 levels. Without widespread DOTS-Plus MDR TB treatment availability, rapid MDR TB diagnosis policies are not as effective, and the prevalence in scenario 2 does not decline as much as in the other scenarios. In contrast, there is a smaller prevalence gap between the base case and scenario 5, where MDR TB has reduced transmission fitness. Since improving MDR TB diagnosis reduces MDR TB cases by preventing transmission, its effect is attenuated in this scenario.

Figure S22: The Effect of Implementing Rapid MDR TB Diagnosis Policies in 2017 Under Various Scenarios. The scenarios are described in the Figure S20 description.

*
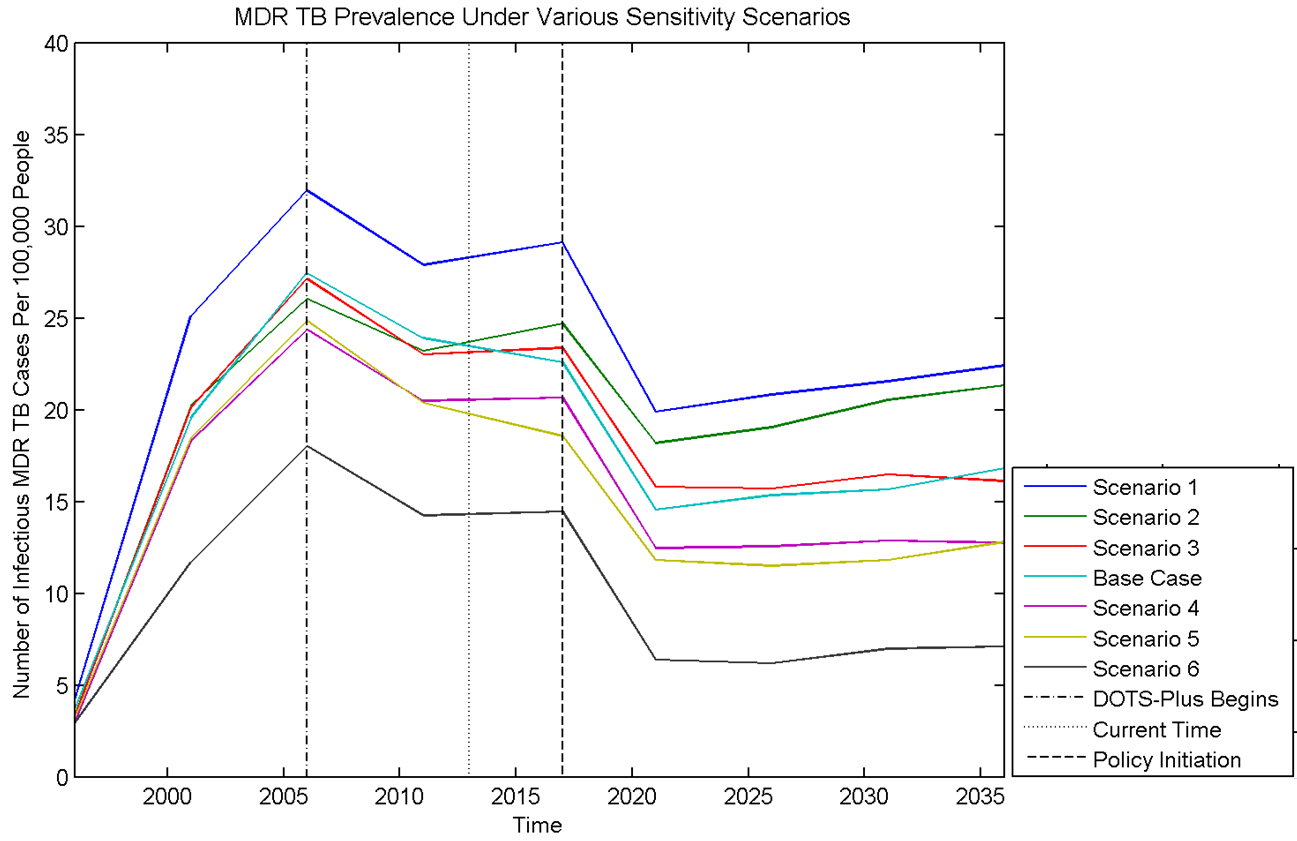
*


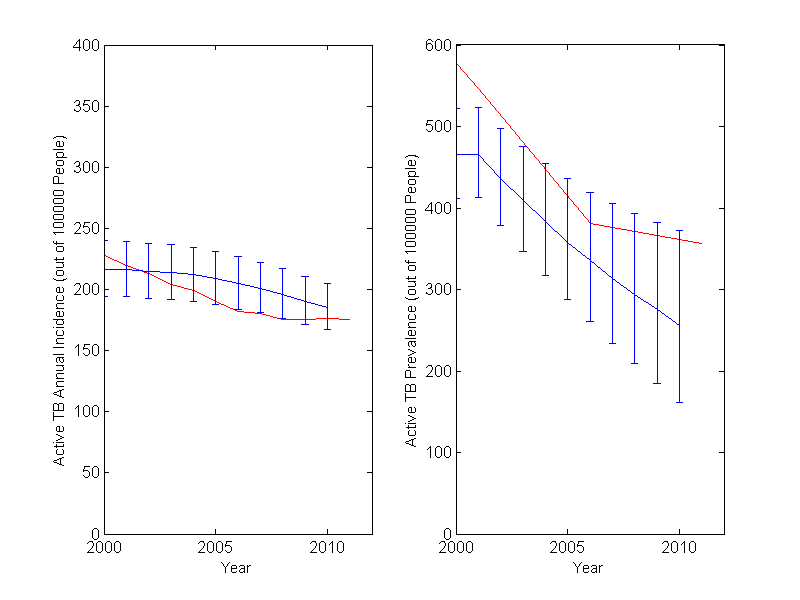
*Effect of Alternative Activation Parameters*

Some uncertainty exists around the timing of activation after TB infection, so we varied the relative rates of activation according to two major studies in the literature. The base case uses activation parameters given by Horsburgh [11], which provide finer age-stratifications than the activation inputs derived from the model in Vynnicky [12]. Since overall activation levels are uncertain in the literature, and we had previously calibrated the overall activation rate for the base case (see Calibration section above), we repeated this procedure for the alternative activation inputs. Calibration results are within uncertainty ranges of empirical data. In particular incidence, prevalence, and demographic characteristics are similar to those reported in the literature for the calibration period (Figure S23 and Table S4 below).

Figure S23: Incidence and Prevalence from the Recalibrated Model (red) Compared to WHO Reported Figures (blue)

**Table S4: Demographic, TB, and Treatment Validation Results With Alternative Activation Inputs**

|  | **Empirical Estimate (95% CI, if available)** | **Simulation Estimate (95% CI)** | **Source** |
| --- | --- | --- | --- |
| **Demographics** |  |  |  |
| Male life expectancy, 1990 (years) | 57.2 | 57.2 (56.4 - 58.0) | [2] |
| Female life expectancy, 1990 (years) | 57.9 | 57.0 (56.3 - 57.8) | [2] |
|  |  |  |  |
| **Tuberculosis** |  |  |  |
| Life expectancy post TB activation   without treatment (years): | ~3 years | 3.23 (3.14 - 3.33) | [33] |
| Lifetime fraction of latent infections that  activate: | 0.10 - 0.20 | 0.11 (0.10 - 0.11) | [11] |
|  |  |  |  |
| **Treatment** |  |  |  |
| Average delay from symptom onset to RNTCP treatment (months) | 2 to 7 | 7.7 | [14,17–19,34] |

We found that the base case results are robust to the use of the alternative set of activation inputs, and time trends remain the same, with an increase in the relative contribution of transmission-generated MDR TB over time, declining effectiveness with delays in non-MDR treatment improvement, and constant effectiveness of MDR diagnosis improvement policies (Figures S24 and S25 below).


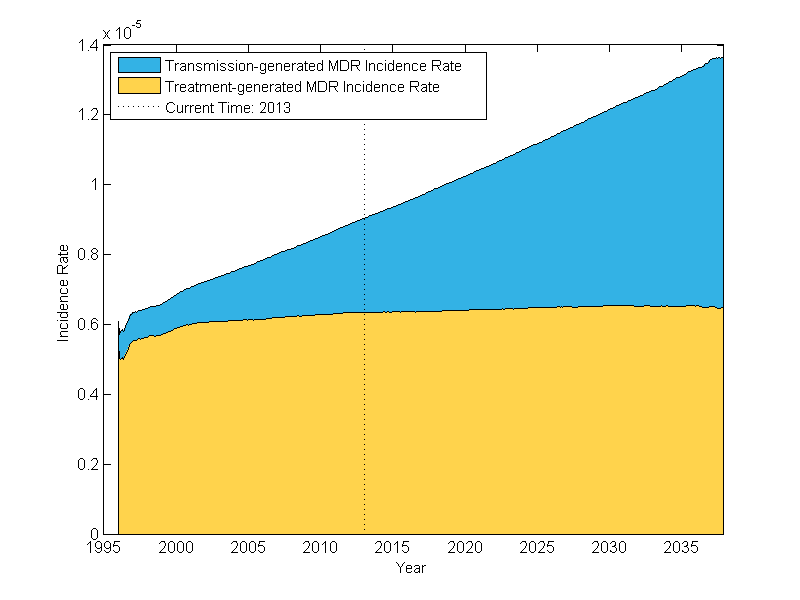


Figure S24: Transmission-Generated MDR TB Incidence is Growing Relative to Treatment-Generated MDR TB Using the Alternative Activation Inputs.


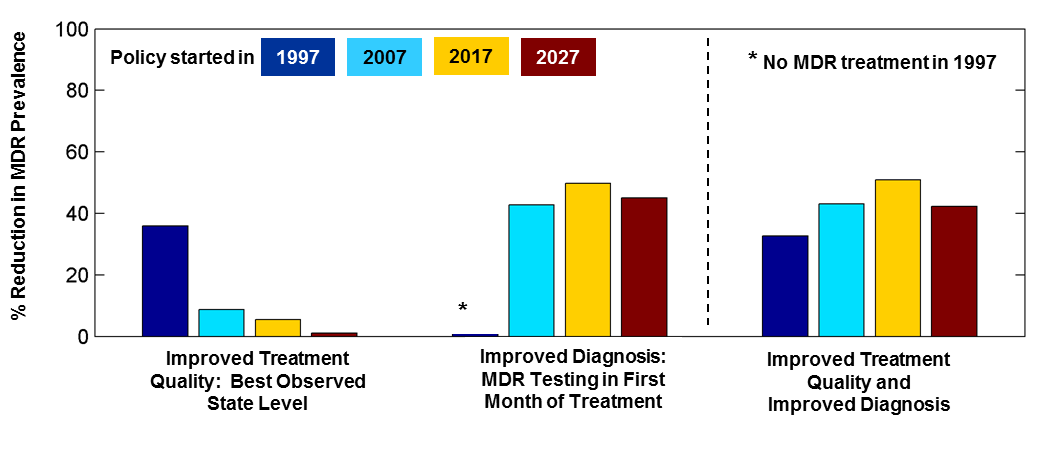


Figure S25: Effects of Delays in Non-MDR TB Treatment Quality Improvements.

**Model Inputs Table**

Table S5 shows values for all model parameters. All values are monthly probabilities, unless otherwise noted. Values given by age indicate that the value used for individuals from the given age to the next age provided; if no additional values are given the value is used for all remaining ages.

- *Table S5.1: Initial Cohort Parameters for Model Burn-In* provides the initial model inputs. This population was then subject to the 130-year burn-in process described in the Model Overview section, which produced a population that matched a variety of demographic and epidemiological data in India 1996 (see Internal and Face Validation section).
- *Table S5.2: Population Growth* provides the model inputs used to generate the birth rate and increase the model population to reflect population growth in India over the calibration and analysis periods.
- *Table S5.3: Mortality* provides baseline and untreated active TB mortality inputs by age and sex.
- *Table S5.4: TB Activation* shows activation input parameters.
- *Table S55: Sputum Smear Test Characteristics* and *Table S5.6: MDR Testing Parameters* provides the test characteristics used in the model. All non-MDR diagnosis uses three sputum smear tests.
- Indicated parameters in *Table S5.7: Treatment Uptake* were calibrated to reported RNTCP and patient demographics (see Calibration section).
- *Table S5.8: Treatment Parameters* provides the treatment coverage and base case and scenario case inputs for death, default, and failure in Cat I/III, Cat II, and Cat IV treatment. Calculations for generating the sex and age stratifications are detailed in the Treatment section above.
- *Table S5.9: Transmission Parameters* provides the contact matrix modified from Mossong. [6] Details on modification are provided in the Transmission section above. The effective contact rate was calibrated (see Calibration section) to generate transmission that would result in TB prevalence and incidence consistent with WHO data.

**Table S5: Values for Model Inputs**

| **Description** | | | | | **Age**  **(years)** | | | **Values** | | |  | | | | **Source** | | |
| --- | --- | --- | --- | --- | --- | --- | --- | --- | --- | --- | --- | --- | --- | --- | --- | --- | --- |
| **S5.1 Initial Cohort Parameters for Model Burn-in** | | | | |  | | |  | | |  | | | |  | | |
| Proportion of population below given age | | | | | 0 | | | 0.073 | | |  | | | | [2] | | |
|  | | | | | 1 | | | 0.141 | | |  | | | |  | | |
|  | | | | | 5 | | | 0.208 | | |  | | | |  | | |
|  | | | | | 10 | | | 0.273 | | |  | | | |  | | |
|  | | | | | 15 | | | 0.339 | | |  | | | |  | | |
|  | | | | | 20 | | | 0.403 | | |  | | | |  | | |
|  | | | | | 25 | | | 0.467 | | |  | | | |  | | |
|  | | | | | 30 | | | 0.529 | | |  | | | |  | | |
|  | | | | | 35 | | | 0.591 | | |  | | | |  | | |
|  | | | | | 40 | | | 0.651 | | |  | | | |  | | |
|  | | | | | 45 | | | 0.710 | | |  | | | |  | | |
|  | | | | | 50 | | | 0.766 | | |  | | | |  | | |
|  | | | | | 55 | | | 0.819 | | |  | | | |  | | |
|  | | | | | 60 | | | 0.868 | | |  | | | |  | | |
|  | | | | | 65 | | | 0.911 | | |  | | | |  | | |
|  | | | | | 70 | | | 0.945 | | |  | | | |  | | |
|  | | | | | 75 | | | 0.970 | | |  | | | |  | | |
|  | | | | | 80 | | | 0.986 | | |  | | | |  | | |
|  | | | | | 85 | | | 0.994 | | |  | | | |  | | |
|  | | | | | 90 | | | 0.998 | | |  | | | |  | | |
|  | | | | | 95 | | | 1.000 | | |  | | | |  | | |
|  | | | | | 100 | | | 1.000 | | |  | | | |  | | |
|  | | | | |  | | |  | | |  | | | |  | | |
| Proportion of each age group that is male | | | | | 0 | | | 0.500 | | |  | | | | [2] | | |
|  | | | | | 1 | | | 0.500 | | |  | | | |  | | |
|  | | | | | 5 | | | 0.503 | | |  | | | |  | | |
|  | | | | | 10 | | | 0.504 | | |  | | | |  | | |
|  | | | | | 15 | | | 0.504 | | |  | | | |  | | |
|  | | | | | 20 | | | 0.505 | | |  | | | |  | | |
|  | | | | | 25 | | | 0.505 | | |  | | | |  | | |
|  | | | | | 30 | | | 0.505 | | |  | | | |  | | |
|  | | | | | 35 | | | 0.504 | | |  | | | |  | | |
|  | | | | | 40 | | | 0.502 | | |  | | | |  | | |
|  | | | | | 45 | | | 0.499 | | |  | | | |  | | |
|  | | | | | 50 | | | 0.495 | | |  | | | |  | | |
|  | | | | | 55 | | | 0.489 | | |  | | | |  | | |
|  | | | | | 60 | | | 0.481 | | |  | | | |  | | |
|  | | | | | 65 | | | 0.470 | | |  | | | |  | | |
|  | | | | | 70 | | | 0.456 | | |  | | | |  | | |
|  | | | | | 75 | | | 0.443 | | |  | | | |  | | |
|  | | | | | 80 | | | 0.421 | | |  | | | |  | | |
|  | | | | | 85 | | | 0.400 | | |  | | | |  | | |
|  | | | | | 90 | | | 0.380 | | |  | | | |  | | |
|  | | | | | 95 | | | 0.362 | | |  | | | |  | | |
|  | | | | | 100 | | | 0.348 | | |  | | | |  | | |
|  | | | | |  | | |  | | |  | | | |  | | |
|  | | | | |  | | | Male | | | Female | | | |  | | |
| Proportion of total population that lives in urban areas | | | | | All Ages | | | 0.283 | | | 0.273 | | | | [38] | | |
|  | | | | |  | | |  | | |  | | | |  | | |
| Prevalence of latent TB in the initial cohort | | | | | 0 | | | 0.040 | | |  | | | | Calibrated, [3] | | |
|  | | | | | 5 | | | 0.080 | | |  | | | |  | | |
|  | | | | | 10 | | | 0.080 | | |  | | | |  | | |
|  | | | | | 15+ | | | 0.090 | | |  | | | |  | | |
|  | | | | |  | | |  | | |  | | | |  | | |
| Prevalence of active TB in initial cohort | | | | |  | | | Male | | | Female | | | | [39] | | |
|  | | | | | 0 | | | 0 | | | 0 | | | |  | | |
|  | | | | | 15 | | | 0.00249 | | | 0.00047 | | | |  | | |
|  | | | | | 25 | | | 0.00693 | | | 0.00223 | | | |  | | |
|  | | | | | 35 | | | 0.01527 | | | 0.00303 | | | |  | | |
|  | | | | | 45 | | | 0.02681 | | | 0.00279 | | | |  | | |
|  | | | | | 55 | | | 0.04313 | | | 0.00681 | | | |  | | |
|  | | | | | 65+ | | | 0.04534 | | | 0.00605 | | | |  | | |
|  | | | | |  | | |  | | |  | | | |  | | |
| Proportion of total active TB individuals who have active MDR in 1996 | | | | |  | | | 0.005 | | |  | | | | [40] | | |
| Proportion of total latent TB individuals who have latent MDR in 1996 | | | | |  | | | 0.005 | | |  | | | | [40] | | |
|  | | | | |  | | |  | | |  | | | |  | | |
| **S5.2 Population Growth** | | | | | Year | | | Value | | |  | | | |  | | |
| Annual percentage population growth | | | | | 1995 | | | 0.02076 | | |  | | | | [1] | | |
|  | | | | | 2000 | | | 0.01854 | | |  | | | |  | | |
|  | | | | | 2005 | | | 0.01635 | | |  | | | |  | | |
|  | | | | | 2010 | | | 0.01484 | | |  | | | |  | | |
|  | | | | | 2015 | | | 0.01365 | | |  | | | |  | | |
|  | | | | | 2020 | | | 0.01203 | | |  | | | |  | | |
|  | | | | | 2025 | | | 0.01039 | | |  | | | |  | | |
|  | | | | | 2030 | | | 0.00885 | | |  | | | |  | | |
|  | | | | | 2035 | | | 0.00739 | | |  | | | |  | | |
|  | | | | | 2040 | | | 0.00598 | | |  | | | |  | | |
|  | | | | |  | | |  | | |  | | | |  | | |
| Population in India including future UN projections | | | | Year | | # of people | | | | |  | [1] | | |  |  |  |
|  | | | | 1950 | | 371,857,000 | | | | |  |  | | |  |  |  |
|  | | | | 1955 | | 406,374,000 | | | | |  |  | | |  |  |  |
|  | | | | 1960 | | 447,844,000 | | | | |  |  | | |  |  |  |
|  | | | | 1965 | | 496,400,000 | | | | |  |  | | |  |  |  |
|  | | | | 1970 | | 553,874,000 | | | | |  |  | | |  |  |  |
|  | | | | 1975 | | 622,097,000 | | | | |  |  | | |  |  |  |
|  | | | | 1980 | | 700,059,000 | | | | |  |  | | |  |  |  |
|  | | | | 1985 | | 784,491,000 | | | | |  |  | | |  |  |  |
|  | | | | 1990 | | 873,785,000 | | | | |  |  | | |  |  |  |
|  | | | | 1995 | | 964,486,000 | | | | |  |  | | |  |  |  |
|  | | | | 2000 | | 1,053,898,000 | | | | |  |  | | |  |  |  |
|  | | | | 2005 | | 1,140,043,000 | | | | |  |  | | |  |  |  |
|  | | | | 2010 | | 1,224,614,000 | | | | |  |  | | |  |  |  |
|  | | | | 2015 | | 1,308,221,000 | | | | |  |  | | |  |  |  |
|  | | | | 2020 | | 1,386,909,000 | | | | |  |  | | |  |  |  |
|  | | | | 2025 | | 1,458,958,000 | | | | |  |  | | |  |  |  |
|  | | | | 2030 | | 1,523,482,000 | | | | |  |  | | |  |  |  |
|  | | | | 2035 | | 1,579,802,000 | | | | |  |  | | |  |  |  |
|  | | | | 2040 | | 1,627,029,000 | | | | |  |  | | |  |  |  |
|  | | | | 2045 | | 1,664,519,000 | | | | |  |  | | |  |  |  |
|  | | | | 2050 | | 1,692,008,000 | | | | |  |  | | |  |  |  |
|  | |  |  |  | | | | |  |  | | | | | |  |  |
| **S5.3 Mortality** |  | |  | |  | | |  | | | | |  | |  |  |  |
| Mortality probability, males by age | | | Year 1990 | | Year 2000 | | | Year 2009 | | | | | [2] | |  |  |  |
|  | 0 | | 0.0073 | | 0.0058 | | | 0.0043 | | | | |  | |  |  |  |
|  | 1 | | 0.0006 | | 0.0005 | | | 0.0003 | | | | |  | |  |  |  |
|  | 5 | | 0.0002 | | 0.0002 | | | 0.0001 | | | | |  | |  |  |  |
|  | 10 | | 0.0001 | | 0.0001 | | | 0.0001 | | | | |  | |  |  |  |
|  | 15 | | 0.0002 | | 0.0002 | | | 0.0001 | | | | |  | |  |  |  |
|  | 20 | | 0.0002 | | 0.0002 | | | 0.0002 | | | | |  | |  |  |  |
|  | 25 | | 0.0003 | | 0.0003 | | | 0.0002 | | | | |  | |  |  |  |
|  | 30 | | 0.0003 | | 0.0003 | | | 0.0003 | | | | |  | |  |  |  |
|  | 35 | | 0.0004 | | 0.0004 | | | 0.0004 | | | | |  | |  |  |  |
|  | 40 | | 0.0005 | | 0.0005 | | | 0.0005 | | | | |  | |  |  |  |
|  | 45 | | 0.0009 | | 0.0008 | | | 0.0007 | | | | |  | |  |  |  |
|  | 50 | | 0.0013 | | 0.0011 | | | 0.001 | | | | |  | |  |  |  |
|  | 55 | | 0.0020 | | 0.0018 | | | 0.0014 | | | | |  | |  |  |  |
|  | 60 | | 0.0030 | | 0.0026 | | | 0.0024 | | | | |  | |  |  |  |
|  | 65 | | 0.0045 | | 0.0041 | | | 0.0036 | | | | |  | |  |  |  |
|  | 70 | | 0.0069 | | 0.0055 | | | 0.0057 | | | | |  | |  |  |  |
|  | 75 | | 0.0096 | | 0.0085 | | | 0.0081 | | | | |  | |  |  |  |
|  | 80 | | 0.0132 | | 0.0104 | | | 0.0116 | | | | |  | |  |  |  |
|  | 85 | | 0.0181 | | 0.0134 | | | 0.0164 | | | | |  | |  |  |  |
|  | 90 | | 0.0247 | | 0.0182 | | | 0.0231 | | | | |  | |  |  |  |
|  | 95 | | 0.0337 | | 0.0260 | | | 0.0323 | | | | |  | |  |  |  |
|  | 99 | | 1 | | 1 | | | 1 | | | | |  | |  |  |  |
|  |  | |  | |  | | |  | | | | |  | |  |  |  |
| Mortality probability, females by age |  | | Year 1990 | | Year 2000 | | | Year 2009 | | | | | [2] | |  |  |  |
|  | 0 | | 0.0075 | | 0.0060 | | | 0.0044 | | | | |  | |  |  |  |
|  | 1 | | 0.0010 | | 0.0007 | | | 0.0004 | | | | |  | |  |  |  |
|  | 5 | | 0.0003 | | 0.0002 | | | 0.0001 | | | | |  | |  |  |  |
|  | 10 | | 0.0001 | | 0.0001 | | | 0.0001 | | | | |  | |  |  |  |
|  | 15 | | 0.0002 | | 0.0002 | | | 0.0001 | | | | |  | |  |  |  |
|  | 20 | | 0.0003 | | 0.0003 | | | 0.0002 | | | | |  | |  |  |  |
|  | 25 | | 0.0003 | | 0.0003 | | | 0.0002 | | | | |  | |  |  |  |
|  | 30 | | 0.0003 | | 0.0003 | | | 0.0002 | | | | |  | |  |  |  |
|  | 35 | | 0.0003 | | 0.0003 | | | 0.0002 | | | | |  | |  |  |  |
|  | 40 | | 0.0004 | | 0.0003 | | | 0.0003 | | | | |  | |  |  |  |
|  | 45 | | 0.0006 | | 0.0005 | | | 0.0004 | | | | |  | |  |  |  |
|  | 50 | | 0.0009 | | 0.0008 | | | 0.0006 | | | | |  | |  |  |  |
|  | 55 | | 0.0014 | | 0.0013 | | | 0.0010 | | | | |  | |  |  |  |
|  | 60 | | 0.0022 | | 0.0018 | | | 0.0018 | | | | |  | |  |  |  |
|  | 65 | | 0.0036 | | 0.0032 | | | 0.0027 | | | | |  | |  |  |  |
|  | 70 | | 0.0059 | | 0.0046 | | | 0.0047 | | | | |  | |  |  |  |
|  | 75 | | 0.0079 | | 0.0071 | | | 0.0065 | | | | |  | |  |  |  |
|  | 80 | | 0.0124 | | 0.0091 | | | 0.0100 | | | | |  | |  |  |  |
|  | 85 | | 0.0186 | | 0.0122 | | | 0.0150 | | | | |  | |  |  |  |
|  | 90 | | 0.0267 | | 0.0170 | | | 0.0220 | | | | |  | |  |  |  |
|  | 95 | | 0.0367 | | 0.0249 | | | 0.0315 | | | | |  | |  |  |  |
|  | 99 | | 1 | | 1 | | | 1 | | | | |  | |  |  |  |
|  |  | |  | |  | | |  | | | | |  | |  |  |  |
| Untreated TB mortality probability, males by age |  | | Year 1990 | | Year 2000 | | | Year 2009 | | | | | [3] | |  |  |  |
|  | 0 | | 0.0318 | | 0.0304 | | | 0.0289 | | | | |  | |  |  |  |
|  | 1 | | 0.0253 | | 0.0251 | | | 0.0249 | | | | |  | |  |  |  |
|  | 5 | | 0.0249 | | 0.0248 | | | 0.0248 | | | | |  | |  |  |  |
|  | 10 | | 0.0248 | | 0.0248 | | | 0.0248 | | | | |  | |  |  |  |
|  | 15 | | 0.0255 | | 0.0255 | | | 0.0255 | | | | |  | |  |  |  |
|  | 20 | | 0.0268 | | 0.0268 | | | 0.0268 | | | | |  | |  |  |  |
|  | 25 | | 0.0275 | | 0.0275 | | | 0.0275 | | | | |  | |  |  |  |
|  | 30 | | 0.0283 | | 0.0283 | | | 0.0283 | | | | |  | |  |  |  |
|  | 35 | | 0.0288 | | 0.0288 | | | 0.0288 | | | | |  | |  |  |  |
|  | 40 | | 0.0294 | | 0.0294 | | | 0.0294 | | | | |  | |  |  |  |
|  | 45 | | 0.0299 | | 0.0298 | | | 0.0297 | | | | |  | |  |  |  |
|  | 50 | | 0.0304 | | 0.0303 | | | 0.0302 | | | | |  | |  |  |  |
|  | 55 | | 0.0308 | | 0.0307 | | | 0.0303 | | | | |  | |  |  |  |
|  | 60 | | 0.0315 | | 0.0311 | | | 0.0310 | | | | |  | |  |  |  |
|  | 65 | | 0.0329 | | 0.0325 | | | 0.0320 | | | | |  | |  |  |  |
|  | 70 | | 0.0351 | | 0.0338 | | | 0.0340 | | | | |  | |  |  |  |
|  | 75 | | 0.0375 | | 0.0364 | | | 0.0360 | | | | |  | |  |  |  |
|  | 80 | | 0.0405 | | 0.0378 | | | 0.0390 | | | | |  | |  |  |  |
|  | 85 | | 0.0453 | | 0.0408 | | | 0.0438 | | | | |  | |  |  |  |
|  | 90 | | 0.0518 | | 0.0455 | | | 0.0503 | | | | |  | |  |  |  |
|  | 95 | | 0.0592 | | 0.0517 | | | 0.0579 | | | | |  | |  |  |  |
|  | 99 | | 1 | | 1 | | | 1 | | | | |  | |  |  |  |
|  |  | |  | |  | | |  | | | | |  | |  |  |  |
| Mortality probability, females by age |  | | Year 1990 | | Year 2000 | | | Year 2009 | | | | |  | |  |  |  |
|  | 0 | | 0.032 | | 0.0305 | | | 0.029 | | | | |  | |  |  |  |
|  | 1 | | 0.0256 | | 0.0254 | | | 0.0251 | | | | |  | |  |  |  |
|  | 5 | | 0.025 | | 0.0249 | | | 0.0248 | | | | |  | |  |  |  |
|  | 10 | | 0.0248 | | 0.0248 | | | 0.0248 | | | | |  | |  |  |  |
|  | 15 | | 0.0250 | | 0.0249 | | | 0.0248 | | | | |  | |  |  |  |
|  | 20 | | 0.0251 | | 0.0250 | | | 0.0249 | | | | |  | |  |  |  |
|  | 25 | | 0.0251 | | 0.0251 | | | 0.0250 | | | | |  | |  |  |  |
|  | 30 | | 0.0252 | | 0.0252 | | | 0.0251 | | | | |  | |  |  |  |
|  | 35 | | 0.0254 | | 0.0253 | | | 0.0253 | | | | |  | |  |  |  |
|  | 40 | | 0.0255 | | 0.0255 | | | 0.0254 | | | | |  | |  |  |  |
|  | 45 | | 0.0258 | | 0.0257 | | | 0.0256 | | | | |  | |  |  |  |
|  | 50 | | 0.0262 | | 0.0261 | | | 0.0259 | | | | |  | |  |  |  |
|  | 55 | | 0.0269 | | 0.0268 | | | 0.0265 | | | | |  | |  |  |  |
|  | 60 | | 0.0279 | | 0.0275 | | | 0.0274 | | | | |  | |  |  |  |
|  | 65 | | 0.0294 | | 0.0289 | | | 0.0284 | | | | |  | |  |  |  |
|  | 70 | | 0.0317 | | 0.0304 | | | 0.0305 | | | | |  | |  |  |  |
|  | 75 | | 0.0335 | | 0.0328 | | | 0.0322 | | | | |  | |  |  |  |
|  | 80 | | 0.0379 | | 0.0347 | | | 0.0356 | | | | |  | |  |  |  |
|  | 85 | | 0.0444 | | 0.0381 | | | 0.0409 | | | | |  | |  |  |  |
|  | 90 | | 0.0527 | | 0.0433 | | | 0.0481 | | | | |  | |  |  |  |
|  | 95 | | 0.0618 | | 0.0502 | | | 0.0567 | | | | |  | |  |  |  |
|  | 99 | | 1 | | 1 | | | 1 | | | | |  | |  |  |  |
|  |  | |  | |  | | |  | | | | |  | |  |  |  |
| **S5.4 TB Activation** |  | |  | |  | | |  | | | | |  | |  |  |  |
| TB activation level (base case) | Age | | Fast | | Slow | | |  | | | | | [11] | |  |  |  |
|  | 0 | | 0.001000 | | 0.000432 | | |  | | | | |  | |  |  |  |
|  | 1 | | 0.001000 | | 0.000432 | | |  | | | | |  | |  |  |  |
|  | 5 | | 0.001000 | | 0.000432 | | |  | | | | |  | |  |  |  |
|  | 10 | | 0.000200 | | 0.000252 | | |  | | | | |  | |  |  |  |
|  | 15 | | 0.000200 | | 0.000261 | | |  | | | | |  | |  |  |  |
|  | 20 | | 0.001100 | | 0.000380 | | |  | | | | |  | |  |  |  |
|  | 25 | | 0.001200 | | 0.000395 | | |  | | | | |  | |  |  |  |
|  | 30 | | 0.001200 | | 0.000410 | | |  | | | | |  | |  |  |  |
|  | 35 | | 0.000900 | | 0.000265 | | |  | | | | |  | |  |  |  |
|  | 40 | | 0.001000 | | 0.000272 | | |  | | | | |  | |  |  |  |
|  | 45 | | 0.001000 | | 0.000275 | | |  | | | | |  | |  |  |  |
|  | 50 | | 0.001000 | | 0.000278 | | |  | | | | |  | |  |  |  |
|  | 55 | | 0.000400 | | 0.000276 | | |  | | | | |  | |  |  |  |
|  | 60 | | 0.000400 | | 0.000274 | | |  | | | | |  | |  |  |  |
|  | 65 | | 0.000400 | | 0.000273 | | |  | | | | |  | |  |  |  |
|  | 70 | | 0.000400 | | 0.000273 | | |  | | | | |  | |  |  |  |
|  | 75 | | 0.000400 | | 0.000267 | | |  | | | | |  | |  |  |  |
|  | 80 | | 0.000400 | | 0.000262 | | |  | | | | |  | |  |  |  |
|  | 85 | | 0.000400 | | 0.000268 | | |  | | | | |  | |  |  |  |
|  | 90 | | 0.000400 | | 0.000275 | | |  | | | | |  | |  |  |  |
|  | 95 | | 0.000400 | | 0.000251 | | |  | | | | |  | |  |  |  |
|  | 99 | | 0.000300 | | 0.000229 | | |  | | | | |  | |  |  |  |
|  |  | |  | |  | | |  | | | | |  | |  |  |  |
| Scaling factor for activation: multiplies activation probabilities (for less and more than 2 years ago) at all ages (final products listed above) | All Ages | | 2.16 | |  | | | Calibrated | | | | |  | |  |  |  |
|  |  | |  | |  | | |  | | | | |  | |  |  |  |
| TB activation level (sensitivity analysis scenario) | | | Fast | | Slow | | |  | | | | |  | |  |  |  |
|  | 0 | | 0.00009000 | | 0 | | |  | | | | | [12] | |  |  |  |
|  | 1 | | 0.00009000 | | 0 | | |  | | | | |  | |  |  |  |
|  | 5 | | 0.00009000 | | 0 | | |  | | | | |  | |  |  |  |
|  | 10 | | 0.00020400 | | 0.00000163 | | |  | | | | |  | |  |  |  |
|  | 15 | | 0.00021200 | | 0.00000169 | | |  | | | | |  | |  |  |  |
|  | 20 | | 0.00035800 | | 0.00000360 | | |  | | | | |  | |  |  |  |
|  | 25 | | 0.00037200 | | 0.00000374 | | |  | | | | |  | |  |  |  |
|  | 30 | | 0.00038600 | | 0.00000388 | | |  | | | | |  | |  |  |  |
|  | 35 | | 0.00039600 | | 0.00000398 | | |  | | | | |  | |  |  |  |
|  | 40 | | 0.00040600 | | 0.00000408 | | |  | | | | |  | |  |  |  |
|  | 45 | | 0.00041000 | | 0.00000412 | | |  | | | | |  | |  |  |  |
|  | 50 | | 0.00041500 | | 0.00000417 | | |  | | | | |  | |  |  |  |
|  | 55 | | 0.00041200 | | 0.00000414 | | |  | | | | |  | |  |  |  |
|  | 60 | | 0.00040900 | | 0.00000411 | | |  | | | | |  | |  |  |  |
|  | 65 | | 0.00040800 | | 0.00000410 | | |  | | | | |  | |  |  |  |
|  | 70 | | 0.00040700 | | 0.00000409 | | |  | | | | |  | |  |  |  |
|  | 75 | | 0.00039900 | | 0.00000401 | | |  | | | | |  | |  |  |  |
|  | 80 | | 0.00039100 | | 0.00000393 | | |  | | | | |  | |  |  |  |
|  | 85 | | 0.00040000 | | 0.00000402 | | |  | | | | |  | |  |  |  |
|  | 90 | | 0.00041000 | | 0.00000412 | | |  | | | | |  | |  |  |  |
|  | 95 | | 0.00037500 | | 0.00000377 | | |  | | | | |  | |  |  |  |
|  | 99 | | 0.00034100 | | 0.00000343 | | |  | | | | |  | |  |  |  |
|  |  | |  | |  | | |  | | | | |  | |  |  |  |
| Scaling factor for activation: multiplies activation probabilities (for less and more than 2 years ago) at all ages (listed above) | All Ages | | 3.3 | |  | | | Calibrated | | | | |  | |  |  |  |

| **S5.5 Sputum Smear Test Characteristics** |  |  |  |  |
| --- | --- | --- | --- | --- |
| Test characteristics for 3 sputum smears combined for active pulmonary TB |  |  |  | [16] |
| Sensitivity | All Ages | 60% |  |  |
| Specificity | All Ages | 100% |  |  |
|  |  |  |  |  |
| **S5.6 MDR Testing Parameters** |  |  |  |  |
| Test characteristics of MDR testing using  Löwenstein-Jensen culture | | |  | Presumptive  Gold |
| Sensitivity | All Ages | 100% |  | Standard |
| Specificity | All Ages | 100% |  |  |
|  |  |  |  |  |
| Time required before patient is notified of DST results | All Ages | 6 months |  | [41] |

| **S5.7 Treatment Uptake Parameters** |  |  |  |
| --- | --- | --- | --- |
| Probability of being tested for TB, no prior treatment | All Ages | 0.000850 | Calibrated |
| Probability of being tested for TB, prior treatment | All Ages | 0.001275 | Calibrated |
| Probability of seeking RNTCP treatment, if TB active | All Ages | 0.600000 | [42] |
| Probability of seeking private treatment, if TB active  and not had RNTCP before | All Ages | 0.570000 | [14] |

| Overall probability of receiving RNTCP treatment if never had treatment before, given treatment is available | Age | Males | Females | Calibrated, [21], [42] |
| --- | --- | --- | --- | --- |
|  | 0 | 0.0968 | 0.0402 |  |
|  | 20 | 0.1254 | 0.0477 |  |
|  | 30 | 0.1800 | 0.0861 |  |
|  | 40 | 0.3743 | 0.1033 |  |
|  | 50 | 0.4505 | 0.1244 |  |
|  | 60 | 0.6000 | 0.1158 |  |
|  | 70 | 0.3346 | 0.0326 |  |
|  |  |  |  |  |
| Overall probability of receiving RNTCP treatment if had treatment before, given treatment is available | 0 | 0.2178 | 0.0905 | Calibrated, [21], [42] |
|  | 20 | 0.2821 | 0.1073 |  |
|  | 30 | 0.4051 | 0.1939 |  |
|  | 40 | 0.6000 | 0.2325 |  |
|  | 50 | 0.6000 | 0.2799 |  |
|  | 60 | 0.6000 | 0.2606 |  |
|  | 70 | 0.6000 | 0.0734 |  |

| **S5.8 Treatment Parameters** | |  | |  | |  | |  | |
| --- | --- | --- | --- | --- | --- | --- | --- | --- | --- |
| Category I/III treatment (base case values) |  | | Male | | Female | | [20], [21] | |  |
| Probability of death in category I/III treatment | All Ages | | 0.01010 | | 0.01010 | |  | |  |
|  |  | |  | |  | |  | |  |
| Default probability, given not dead | 0 | | 0.02383 | | 0.02421 | |  | |  |
|  | 20 | | 0.02337 | | 0.01780 | |  | |  |
|  | 30 | | 0.02145 | | 0.01767 | |  | |  |
|  | 40 | | 0.01663 | | 0.01532 | |  | |  |
|  | 50 | | 0.02278 | | 0.01246 | |  | |  |
|  | 60 | | 0.02222 | | 0.01151 | |  | |  |
|  | 70+ | | 0.02256 | | 0.02038 | |  | |  |
|  |  | |  | |  | |  | |  |
| Probability of successful treatment, given patient has been in | All Ages | | 0.98 | | 0.98 | |  | |  |
| treatment for required time and not died nor defaulted |  | |  | |  | |  | |  |
|  |  | |  | |  | |  | |  |
| Category I/III treatment (best observed state values) |  | | Male | | Female | | [20], [21] | |  |
| Probability of death in category I/III treatment | All Ages | | 0.00267 | | 0.00267 | |  | |  |
|  |  | |  | |  | |  | |  |
| Default probability, given not dead | 0 | | 0.005103 | | 0.00518 | |  | |  |
|  | 20 | | 0.005003 | | 0.00381 | |  | |  |
|  | 30 | | 0.004593 | | 0.00378 | |  | |  |
|  | 40 | | 0.003560 | | 0.00328 | |  | |  |
|  | 50 | | 0.004877 | | 0.00267 | |  | |  |
|  | 60 | | 0.004757 | | 0.00246 | |  | |  |
|  | 70+ | | 0.004830 | | 0.00436 | |  | |  |
|  |  | |  | |  | |  | |  |
| Probability of successful treatment, given patient has been in | All Ages | | 0.99 | | 0.99 | |  | |  |
| treatment for required time and not died nor defaulted |  | |  | |  | |  | |  |
|  |  | |  | |  | |  | |  |
| Category II treatment (base case values) |  | | Male | | Female | | [20], [21] | |  |
| Probability of death in category II treatment | All Ages | | 0.02600 | | 0.02600 | |  | |  |
|  |  | |  | |  | |  | |  |
| Default probability, given not dead | 0 | | 0.05804 | | 0.05895 | |  | |  |
|  | 20 | | 0.05690 | | 0.04335 | |  | |  |
|  | 30 | | 0.05223 | | 0.04303 | |  | |  |
|  | 40 | | 0.04051 | | 0.03730 | |  | |  |
|  | 50 | | 0.05547 | | 0.03034 | |  | |  |
|  | 60 | | 0.05411 | | 0.02803 | |  | |  |
|  | 70+ | | 0.05493 | | 0.04963 | |  | |  |
|  |  | |  | |  | |  | |  |
| Probability of successful treatment, given patient has been in | All Ages | | 0.94 | | 0.94 | |  | |  |
| treatment for required time and not died nor defaulted |  | |  | |  | |  | |  |
| Probability of testing SS+ at month 4 of cat. II treatment for | 0.57 | |  | |  | | [23] | |  |
| non-MDR TB patients |  | |  | |  | |  | |  |
|  |  | |  | |  | |  | |  |
| Category II treatment (best observed state values) |  | | Male | | Female | | [21], [20] | |  |
| Probability of death in category II treatment | All Ages | | 0.01454 | | 0.01454 | |  | |  |
|  |  | |  | |  | |  | |  |
| Default probability, given not dead | 0 | | 0.00827 | | 0.00840 | |  | |  |
|  | 20 | | 0.00811 | | 0.00618 | |  | |  |
|  | 30 | | 0.00744 | | 0.00613 | |  | |  |
|  | 40 | | 0.00577 | | 0.00532 | |  | |  |
|  | 50 | | 0.00791 | | 0.00432 | |  | |  |
|  | 60 | | 0.00771 | | 0.00400 | |  | |  |
|  | 70+ | | 0.00783 | | 0.00707 | |  | |  |
|  |  | |  | |  | |  | |  |
| Probability of successful treatment, given patient has been in | All Ages | | 0.972 | | 0.972 | |  | |  |
| treatment for required time and not died nor defaulted |  | |  | |  | |  | |  |
| Probability of developing MDR TB if default from treatment | All Ages | | 0.242 | |  | | [43] | |  |
| Probability of developing MDR TB if fail from treatment | All Ages | | 0.187 | |  | | [43] | |  |
| Probability of having latent TB after treatment | All Ages | | 0.197 | |  | | [43] | |  |
|  |  | |  | |  | |  | |  |
| Category IV treatment |  | |  | |  | |  | |  |
| Probability of death in category IV treatment | All Ages | | 0.01689 | |  | | [44] | |  |
|  |  | |  | |  | |  | |  |
| Default probability, given not dead | All Ages | | 0.01749 | |  | |  | |  |
|  |  | |  | |  | |  | |  |
| Probability of successful treatment, given patient has been in | All Ages | | 0.73750 | |  | |  | |  |
| treatment for required time and not died nor defaulted |  | |  | |  | |  | |  |
| DOTS treatment scale up over 1996 - 2006 | Year | | Proportion of nation covered | |  | | [30] | |  |
| (linearly interpolated to monthly values between data points) | 1996 | | 0.000 | |  | |  | |  |
|  | 1997 | | 0.075 | |  | |  | |  |
|  | 1998 | | 0.150 | |  | |  | |  |
|  | 1999 | | 0.225 | |  | |  | |  |
|  | 2000 | | 0.300 | |  | |  | |  |
|  | 2001 | | 0.400 | |  | |  | |  |
|  | 2002 | | 0.500 | |  | |  | |  |
|  | 2003 | | 0.731 | |  | |  | |  |
|  | 2004 | | 0.923 | |  | |  | |  |
|  | 2005 | | 0.970 | |  | |  | |  |
|  | 2006 | | 1.000 | |  | |  | |  |

| DOTS-Plus scale up | All Ages | 0.278*exp(0.03109*months since Jan2007 - 1.76) |  | [45] [13] |
| --- | --- | --- | --- | --- |

| **S5.9 Transmission Parameters** |  |  |  |
| --- | --- | --- | --- |
| Average maternal age at birth of first child | All Ages | 17 | [10] |
| Effective contact rate for TB transmission | All Ages | 0.0022 | Calibrated |

| Modified average number of daily contacts (age range of contact on rows, age of individual on columns) |  |  | [6] |
| --- | --- | --- | --- |
|  | | |  |

|  | 0 | 5 | 10 | 15 | 20 | 25 | 30 | 35 | 40 | 45 | 50 | 55 | 60 | 65 | 70+ |
| --- | --- | --- | --- | --- | --- | --- | --- | --- | --- | --- | --- | --- | --- | --- | --- |
| 0 | 0.69 | 0.24 | 0.36 | 0.76 | 0.79 | 0.53 | 0.97 | 0.83 | 0.38 | 0.22 | 0.36 | 0.22 | 0.22 | 0.05 | 0.09 |
| 5 | 0.59 | 1.82 | 0.43 | 0.22 | 0.79 | 0.77 | 0.42 | 1.15 | 1.09 | 0.43 | 0.21 | 0.32 | 0.15 | 0.08 | 0.09 |
| 10 | 0.25 | 0.53 | 1.69 | 0.32 | 0.09 | 1.11 | 0.69 | 0.51 | 0.58 | 0.22 | 0.14 | 0.10 | 0.20 | 0.07 | 0.10 |
| 15 | 0.18 | 0.44 | 0.79 | 1.40 | 0.38 | 0.20 | 0.67 | 0.75 | 0.47 | 0.74 | 0.47 | 0.10 | 0.10 | 0.08 | 0.08 |
| 20 | 0.42 | 0.57 | 0.17 | 0.85 | 1.02 | 0.58 | 0.31 | 0.20 | 0.27 | 0.46 | 0.22 | 0.14 | 0.10 | 0.07 | 0.19 |
| 25 | 0.61 | 0.42 | 0.44 | 0.12 | 0.49 | 0.49 | 0.42 | 0.24 | 0.29 | 0.29 | 0.17 | 0.22 | 0.14 | 0.12 | 0.10 |
| 30 | 0.57 | 0.68 | 0.32 | 0.37 | 0.28 | 0.35 | 0.80 | 0.47 | 0.25 | 0.17 | 0.15 | 0.13 | 0.15 | 0.08 | 0.05 |
| 35 | 0.74 | 0.99 | 0.51 | 0.29 | 0.46 | 0.21 | 0.21 | 0.76 | 0.49 | 0.17 | 0.14 | 0.21 | 0.20 | 0.16 | 0.13 |
| 40 | 0.18 | 0.66 | 0.69 | 0.48 | 0.23 | 0.38 | 0.27 | 0.47 | 0.45 | 0.35 | 0.19 | 0.05 | 0.19 | 0.11 | 0.24 |
| 45 | 0.20 | 0.15 | 0.27 | 0.51 | 0.44 | 0.25 | 0.38 | 0.29 | 0.40 | 0.58 | 0.27 | 0.25 | 0.13 | 0.04 | 0.29 |
| 50 | 0.36 | 0.17 | 0.20 | 0.27 | 0.20 | 0.30 | 0.23 | 0.18 | 0.12 | 0.15 | 0.33 | 0.17 | 0.09 | 0.05 | 0.23 |
| 55 | 0.15 | 0.19 | 0.19 | 0.13 | 0.28 | 0.30 | 0.20 | 0.11 | 0.19 | 0.19 | 0.35 | 0.52 | 0.24 | 0.07 | 0.22 |
| 60 | 0.18 | 0.41 | 0.17 | 0.09 | 0.11 | 0.20 | 0.23 | 0.32 | 0.23 | 0.14 | 0.20 | 0.32 | 0.27 | 0.17 | 0.11 |
| 65 | 0.26 | 0.30 | 0.26 | 0.26 | 0.15 | 0.04 | 0.15 | 0.04 | 0.15 | 0.11 | 0.04 | 0.44 | 0.30 | 0.37 | 0.22 |
| 70+ | 0.07 | 0.07 | 0.17 | 0.37 | 0.00 | 0.03 | 0.07 | 0.03 | 0.30 | 0.20 | 0.07 | 0.10 | 0.13 | 0.43 | 0.70 |

References

1. Population Division of the Department of Economic and Social Affairs of the United Nations Secretariat (2010) World Population Prospects: The 2010 Revision. Available: http://esa.un.org/unpd/wpp/index.htm .

2. World Health Organization (2010) WHO Lifetables for India. Geneva, Switzerland. Available: http://apps.who.int/ghodata/?vid=720.

3. Dye C, Scheele S, Dolin P, Pathania V, Raviglione M (1999) Consensus statement. Global burden of tuberculosis: estimated incidence, prevalence, and mortality by country. JAMA, J Am Med Assoc 282: 677–686.

4. World Health Organization (2011) Global tuberculosis control 2011. Geneva, Switzerland.

5. Keeling M, Rohani P (2007) Modeling infectious diseases in humans and animals. 1st ed. Princeton University Press.

6. Mossong J, Hens N, Jit M, Beutels P, Auranen K, et al. (2008) Social contacts and mixing patterns relevant to the spread of infectious diseases. PLoS Med 5: e74. doi:10.1371/journal.pmed.0050074.

7. Horby P, Pham QT, Hens N, Nguyen TTY, Le QM, et al. (2011) Social contact patterns in Vietnam and implications for the control of infectious diseases. PloS One 6: e16965. doi:10.1371/journal.pone.0016965.

8. World Health Organization (2013) Roadmap for childhood tuberculosis. Geneva, Switzerland.

9. Loeffler A (2003) Pediatric tuberculosis. Seminars in Respiratory Infections 18: 272–291. Available: http://www.ncbi.nlm.nih.gov/pubmed/14679476.

10. Bloom DE, Reddy PH (2011) Age patterns of women at marriage, cohabitation, and first birth in india. Demography 23: 509–523.

11. Horsburgh CR (2004) Priorities for the treatment of latent tuberculosis infection in the United States. N Engl J Med 350: 2060–2067. doi:10.1056/NEJMsa031667.

12. Vynnycky E, Fine PE (1997) The natural history of tuberculosis: the implications of age-dependent risks of disease and the role of reinfection. Epidemiol Infect 119: 183–201.

13. Government of India National DOTS-Plus Committee (2011) Minutes of the Seventh National DOTS-Plus Committee Meeting LRS Institute, New Delhi, 11th and 12th July 2011. Minutes of the Seventh National DOTS-Plus Committee Meeting LRS Institute, New Delhi, 11th and 12th July 2011. Vol. 756. pp. 1–33. Available: http://www.tbcindia.nic.in/pdfs/FinalMinutesof7thNationalDOTSPlusCommitteeMeeting-11-120711.pdf.

14. Kapoor SK, Raman a V, Sachdeva KS, Satyanarayana S (2012) How did the TB patients reach DOTS services in Delhi? A study of patient treatment seeking behavior. PloS One 7: e42458. doi:10.1371/journal.pone.0042458.

15. Dye C (2012) The potential impact of new diagnostic tests on tuberculosis epidemics. Indian J Med Res 135: 737–744.

16. Srikanth P, Kamesh S, Daley P (2009) Bleach optimization of sputum smear microscopy for pulmonary tuberculosis. Indian J Tuberc 56: 174–184.

17. Rajeswari R, Chandrasekaran V, Suhadev M, Sivasubramaniam S, Sudha G, et al. (2002) Factors associated with patient and health system delays in the diagnosis of tuberculosis in South India. Int J Tuberc Lung Dis 6: 789–795. Available: http://www.ncbi.nlm.nih.gov/pubmed/12234134.

18. Selvam JM, Wares F, Perumal M, Gopi PG, Sudha G, et al. (2007) Health-seeking behaviour of new smear-positive TB patients under a DOTS programme in Tamil Nadu, India, 2003. Int J Tuberc Lung Dis 11: 161–167. Available: http://www.ncbi.nlm.nih.gov/pubmed/17263286.

19. Tobgay, KJ, Sarma, PS, Thankappan K (2006) Predictors of treatment delays for tuberculosis in Sikkim. Natl Med J India 19.

20. Central TB Division Directorate General of Health Services and Ministry of Health and Family Welfare (2010) RNTCP performance report, India: Third quarter, 2010. Available: http://www.tbcindia.org.

21. COHESIVE-India Bihar Evaluation of Social Franchising and Telemedicine (2011). Available: www.cohesiveindia.org.

22. Balasubramanian R, Garg R, Santha T, Gopi PG, Subramani R, et al. (2004) Gender disparities in tuberculosis: report from a rural dots programme in south India. Int J Tuberc Lung Dis 8: 323–332. Available: http://www.ncbi.nlm.nih.gov/pubmed/15139471.

23. Santha T, Garg R, Frieden TR, Chandrasekaran V, Subramani R, et al. (2002) Risk factors associated with default, failure and death among tuberculosis patients treated in a DOTS programme in Tiruvallur district, south India, 2000. Int J Tuberc Lung Dis 6: 780–788. Available: http://www.ncbi.nlm.nih.gov/pubmed/12234133.

24. World Health Organization (2010) Multidrug and extensively drug-resistant TB (M/XDR-TB): 2010 global report on surveillance and response. Geneva, Switzerland.

25. Revised National Tuberculosis Control Programme (RNTCP) (2010) Training Module for Medical Practitioners.

26. Arora VK, Sarin R, Singla R, Khalid UK, Mathuria K, et al. (2007) DOTS-Plus for Patients with Multidrug-resistant Tuberculosis in India : Early Results After Three Years. Indian J Chest Dis Allied Sci 49: 75–79.

27. Udwadia ZF, Pinto LM, Uplekar MW (2010) Tuberculosis management by private practitioners in Mumbai, India: has anything changed in two decades? PloS one 5: e12023. doi:10.1371/journal.pone.0012023.

28. Vandan N, Ali M, Prasad R, Kuroiwa C (2009) Assessment of doctors’ knowledge regarding tuberculosis management in Lucknow, India: a public-private sector comparison. Public Health 123: 484–489. doi:10.1016/j.puhe.2009.05.004.

29. Uplekar MW, Shepard DS (1991) Treatment of tuberculosis by private general practitioners in India. Tubercle 72: 284–290.

30. Central TB Division Directorate General of Health Services and Ministry of Health and Family Welfare (2007) TB India 2007: RNTCP Status Report.

31. Chadha VK, Sarin R, Narang P, John KR, Chopra KK, et al. (2013) Trends in the annual risk of tuberculous infection in India. Int J Tuberc Lung Dis 17: 312–319. Available: http://www.ncbi.nlm.nih.gov/pubmed/23321394.

32. Dowdy D, Chaisson R (2009) The persistence of tuberculosis in the age of DOTS: reassessing the effect of case detection. Bulletin of the World Health Organization 87: 296–304. Available: http://www.who.int/bulletin/volumes/87/4/08-054510.pdf. Accessed 22 July 2012.

33. Tiemersma EW, Van der Werf MJ, Borgdorff MW, Williams BG, Nagelkerke NJD (2011) Natural History Of Tuberculosis: Duration And Fatality Of Untreated Pulmonary Tuberculosis In Hiv Negative Patients: A Systematic Review. PloS One 6: e17601. Available: http://www.pubmedcentral.nih.gov/articlerender.fcgi?artid=3070694&tool=pmcentrez&rendertype=abstract. Accessed 16 July 2012.

34. Pantoja A, Floyd K, Unnikrishnan KP, Jitendra R, Padma MR, et al. (2009) Economic evaluation of public-private mix for tuberculosis care and control, India. Part I. Socio-economic profile and costs among tuberculosis patients. Int J Tuberc Lung Dis 13: 698–704.

35. Cohen T, Sommers B, Murray M (2003) The effect of drug resistance on the fitness of Mycobacterium tuberculosis. Lancet Infect Dis 3: 13–21. Available: http://www.sciencedirect.com/science/article/pii/S1473309903004833. Accessed 12 March 2013.

36. Read AF, Day T, Huijben S (2011) The evolution of drug resistance and the curious orthodoxy of aggressive chemotherapy. Proc Natl Acad Sci 108 Suppl: 10871–10877. Available: http://www.pubmedcentral.nih.gov/articlerender.fcgi?artid=3131826&tool=pmcentrez&rendertype=abstract. Accessed 1 March 2013.

37. Gagneux S, Long CD, Small PM, Van T, Schoolnik GK, et al. (2006) The competitive cost of antibiotic resistance in Mycobacterium tuberculosis. Science (New York, NY) 312: 1944–1946. Available: http://www.ncbi.nlm.nih.gov/pubmed/16809538. Accessed 11 March 2013.

38. Census of India (2006) Population Projections For India And States 2001-2026.

39. Gopi PG, Subramani R, Radhakrishna S, Kolappan C, Sadacharam K, et al. (2003) A Baseline Survey Of The Prevalence Of Tuberculosis In A Community In South India At The Commencement Of A Dots Programme. The International Journal of Tuberculosis And Lung Disease 7: 1154–1162. Available: http://www.ncbi.nlm.nih.gov/pubmed/14677890.

40. World Health Organization (2004) Anti-tuberculosis Drug Resistance In The World: Third Global Report. Geneva, Switzerland. doi:10.1111/j.1083-4389.2004.00255-15.x.

41. Singla R, Sarin R, Khalid UK, Mathuria K, Singla N, et al. (2009) Seven-year DOTS-Plus pilot experience in India: results, constraints and issues. Int J Tuberc Lung Dis 13: 976–981.

42. Satyanarayana S, Nair SA, Chadha SS, Shivashankar R, Sharma G, et al. (2011) From where are tuberculosis patients accessing treatment in India? Results from a cross-sectional community based survey of 30 districts. PloS One 6: e24160. doi:10.1371/journal.pone.0024160.

43. Sharma SK, Kumar S, Saha PK, George N, Arora SK, et al. (2011) Prevalence Of Multidrug-resistant Tuberculosis Among Category II Pulmonary Tuberculosis Patients. Indian J Med Res 133: 312–315. Available: http://www.pubmedcentral.nih.gov/articlerender.fcgi?artid=3103157&tool=pmcentrez&rendertype=abstract.

44. Sachdeva KS (2011) Overview of drug resistant TB in India and national scale up of MDR TB diagnosis and treatment TB burden in India. Facing the Reality Of Multidrug-Resistant Tuberculosis: Challenges and Potential Solutions in India, Apr 18-19. New Delhi, India: Indian National Science Academy.

45. Stop TB Partnership (2006) The Global Plan to Stop TB 2006 - 2015: Actions for Life Towards a World Free of Tuberculosis. Geneva, Switzerland.
